# Formation optimization of airborne radar coordinated detection system using an improved Artificial Fish Swarm Algorithm

**DOI:** 10.1038/s41598-023-50521-6

**Published:** 2024-01-02

**Authors:** Tingting Li, Tiankuo Meng, Guanglei Meng, Chenguang Wang, Biao Wang, Mingzhe Zhou, Xingyuan Han

**Affiliations:** 1 Department of Aeronautics and Astronautics, Fudan University, Shanghai, 200433 China; 2 School of Automation, Shenyang Aerospace University, Shenyang, 110136 China; 3 Aviation Science and Technology Key Laboratory of Air Combat System Technology, Shenyang, 110136 China

**Keywords:** Aerospace engineering, Software

## Abstract

In modern air combat, collaborative detection and engagement among multiple aircraft have gradually become a predominant combat approach. In response to the challenges posed by modern stealth aircraft, although their external factors such as coatings significantly reduce the chances of enemy detection, once these stealth aircraft activate their radar systems, they become susceptible to detection. Therefore, an application model has been proposed to mitigate enemy detection of our stealth aircraft through a collaborative approach. The underlying principle involves employing the concept of multi-aircraft collaboration, where the aircraft are divided into transmitters and receivers. The transmitters emit radar waves while the receivers are responsible for receiving these waves. This approach effectively mitigates the increased probability of enemy detection resulting from the activation of our receivers' radar systems. The optimization problem we aim to address is determining the optimal formation configuration for cooperative flight, specifically a formation with a specific configuration that maximizes the detectable range. This optimization problem is known as the configuration optimization problem for Airborne Radar Network with Separate Transmitting and Receiving (ARN-STAR). Existing methods for this problem typically suffer from limitations in either effectiveness or efficiency. To overcome these limitations, we propose an optimized configuration method based on an improved Artificial Fish Swarm Algorithm (IFSA) for ARN-STAR. Firstly, leveraging the distribution characteristics of the target radar wave’s spatial scattering and the concept of dual-radar spatial diversity, we establish a mathematical model and an optimization objective function for ARN-STAR. Secondly, to address efficiency concerns, we optimize the computational process using the IAFS, successfully improving the speed of computation. To address the issue of effectiveness, we introduce adaptive adjustments to the movement step size of the artificial fish and improve the implementation of the three behavioral modes, thereby avoiding local optima and enhancing the accuracy of finding the optimal configuration. Finally, using our self-developed multi-aircraft collaborative simulation platform, we apply the improved AFSA to obtain the optimal formation configuration scheme and compare it with other methods. Simulation results demonstrate that our proposed method effectively solves the problem of finding the optimal formation configuration in multi-aircraft collaborative detection scenarios with “one transmission and multiple receptions.” It overcomes the low computational efficiency associated with traditional methods while maintaining good accuracy. This approach enables the enhancement of overall combat capabilities while ensuring the safety of our aircraft to the greatest extent possible. It should be noted that the scenarios discussed in this study are at the configurational configuration level between UAVs, rather than involving the design of the UAVs combat control system itself.

## Introduction

As modern information technology continues to rapidly advance, the significance of air power in comprehensive national strength has increasingly grown. In modern warfare, the acquisition of air superiority has become a critical factor in determining the outcome of a conflict. However, with the rapid development of stealth technology in combat aircraft, the effective detection of enemy aircraft at greater distances and over larger areas in a covert manner presents a significant challenge to airborne detection technology^1,2^. Airborne detection technology encompasses multiple aspects, of which multi-aircraft coordinated detection stands out as a crucial component. Notably, this technique allows for accurate and efficient identification of enemy locations, guiding subsequent targeted strikes while simultaneously decreasing the likelihood of our critical objectives being detected. Therefore, a comprehensive investigation into the intricacies of multi-aircraft coordinated detection bears substantial military significance and practical value.

In the context of modern stealth aircraft, their reduced detectability due to factors such as coatings and external features is widely acknowledged. However, once these stealth aircraft activate their radar systems, they become highly susceptible to enemy detection. Consequently, the military has introduced an application model aimed at minimizing enemy detection of our stealth aircraft through a collaborative approach involving multiple aircraft.

The underlying principle of this approach relies on the concept of multi-aircraft collaboration, where the aircraft are divided into transmitters and receivers. The transmitters emit radar waves, while the receivers are responsible for receiving these waves. By employing this strategy, we effectively reduce the likelihood of enemy detection resulting from the activation of our receivers' radar systems. Specifically, we position the transmitters at the rear while placing the receivers at the front. Only the transmitters at the rear activate their radar systems, while the receivers at the front solely focus on receiving the radar echoes emitted by the transmitters. As a result, our frontline aircraft remain undetectable during offensive maneuvers since they do not activate their radars.

By adopting this specific formation characterized by a one-to-many reception mode, we aim to optimize the detectable range. Determining the ideal configuration becomes a critical and pressing issue in order to achieve maximum detectability.

In recent years, a considerable number of scholars have undertaken research on airborne detection technology. To optimize the effectiveness of detection, many experts and research teams have carried out extensive work aimed at exploring strategies to enhance the detection performance of radar. Regarding radar signal processing: multipath effect^3^, frequency agility^4,5^, radiation energy reduction constraint method^6^, digital filter^7^, a new method for solving ambiguity^8^, time width of pulse compression^9^, spatio-temporal adaptive processing^10^. The above-mentioned techniques have the potential to enhance the radar’s range and anti-interference capability, significantly strengthening its target processing capabilities. However, these techniques are not without limitations. Lengthy research cycles and inconsistent hardware performance are notable drawbacks. With the rapid development of wireless network technology, modern information technology can provide discrete combat platforms with system-level interconnection information like that of a single platform. This can achieve real-time tactical allocation and cross-platform information sharing. To improve detection efficiency, researchers are shifting their focus from the technical level to the tactical level, namely, collaborative detection.

Airborne radar plays a crucial role in aerial combat capabilities and is increasingly significant. The cooperative detection method, which utilizes a network of airborne radar systems composed of aircraft formations, is regarded as a crucial form of air combat in the future^11^. Regarding the application aspect of Airborne Radar Network (ARN) formation: In terms of security, during the execution of operational missions, radar-equipped airborne platforms form cluster formations, which can be perceived as a spatially separated yet logically integrated virtual detection platform. This detection pattern facilitates complementary advantages among individual entities in the cluster, whereby the destruction of any single node does not affect the normal operation of other nodes, thereby enhancing the survivability of the cluster in complex battlefield environments^12,13^. In terms of detection performance, any member within a group can act as a transmitter while other members function as receivers. Through the utilization of the spatial diversity provided by multi-station airborne radar systems and the optimization of the spatial distribution of transmitters and receivers, enemy targets can be detected more effectively. The existence of networked formations enhances the survivability of transmitters and extends the detection range and operational radius of receivers^14,15^, It satisfies the requirement that the receiver realizes target detection in a covert way. The networked airborne radar has overcome the weakness of insufficient responsiveness of current airborne radar systems to changes on the battlefield, thereby facilitating the maximum operational effectiveness of formation. In the context of radar network applications: Reference^16^ presents radar networking methods and investigates dynamic target detection under various factors. Reference^17^ explores networking of guidance radar for anti-stealth by utilizing multiple sensors to collect target information and fusing data through a data fusion module, fully utilizing the scattering characteristics of targets in different directions and frequency bands to fully collect information possible. The study mainly focuses on filtering fusion algorithms and signal-to-noise ratio weighted fusion algorithms. The effectiveness of radar networking for anti-stealth is validated through specific networking topology simulation. Reference^18^ conducts simulation research on key technologies of radar networking, proposes an improved algorithm based on velocity information for track correlation, and models and analyzes network data processing. Reference^19^ investigates data fusion simulations and relevant algorithms for multiple radar networking, establishes a software platform for multiple radar networking data fusion simulations, and conducts research on optimizing anti-stealth deployment. Reference^20^ studies multi-frequency and multi-radar networking for detecting stealth targets, and explores network resource management problems, providing sensor resource management algorithms that have been validated for effectiveness.

Although the literature reviewed above has extensively investigated radar network formation, we aim to optimize the network formation of multiple radars from the perspective of optimizing the radar deployment angle. In the context of optimizing radar network deployment: Based on the characteristics of target spatial detection and radar cross-section (RCS), we seek to identify an effective deployment strategy for directed targets and a method for filling in the blind spots of the primary station's detection area. In this regard, Reference^21^ conducted research on the cooperative detection of stealth space targets by aviation clusters, but without the use of algorithms, there is significant room for improvement in computational efficiency. Reference^22^ employed the clone immune decision method to enable drone clusters to cooperatively detect and track targets, but accuracy remains an issue. Zhu utilized a binary wolf algorithm to investigate the optimal formation configuration for cooperative detection in aviation clusters, but the computational accuracy was insufficient. Reference^23^ applied a 0–1 resolution matrix to identify blind spots in cluster detection, but the efficiency was low. Reference^24^ optimized the formation configuration of cooperative detection formation using the firework algorithm, yet the effectiveness of the approach was found to be relatively low. After identifying the aforementioned issues, we conducted research on numerous optimization algorithms and eventually selected the Artificial Fish Swarm Algorithm (AFSA) for further improvement. AFSA is a bio-inspired optimization algorithm that simulates the foraging behavior of fish swarms. In recent years, AFSA has garnered considerable attention in the field of optimization due to its effectiveness in addressing complex optimization problems. We discovered several significant pioneering works that have contributed to its development. For instance, in the context of algorithmic hybridization and combination: Li integrates the improved Artificial Fish Swarm Algorithm with continuous segmented Bézier curves for path planning and smoothing of mobile robots^25^. By introducing feasible solutions, a range of step sizes, a dynamic feedback horizon, and adaptive step size, we overcome the low accuracy, convergence, and degradation issues in the traditional algorithm. Regarding the aspects of variant extension and application domains: Guo introduced an enhanced variant of the Artificial Fish Swarm Algorithm: Pair Barracuda Swarm Optimization algorithm^26^. By employing a unique strategy for constructing barracuda pairs and incorporating a support barracuda, PBSO enhances global search capabilities. PBSO effectively mitigates the pitfalls encountered by traditional evolutionary tools in high-dimensional spaces, such as dimensional catastrophes and being trapped in local optima. Through comprehensive analysis and research on the aforementioned issues. In this paper, we optimize the process of solving the optimal formation configuration for air-based radar transmit-receive separation cooperative detection using an improved Artificial Fish Swarm Algorithm, which not only improves the detection efficiency but also enhances the effectiveness of detection.

To meet the practical needs of combat operations, we conducted an analysis of the above-mentioned issues, and proposed a method for optimizing the configuration of cooperative detection formations for air-based radar with separated transmission and reception based on an improved Artificial Fish Swarm Algorithm. We also designed a multi-aircraft cooperative detection simulation software to verify the proposed method. The first step involved analyzing the principle of cooperative detection for air-based radar with separated transmission and reception using target radar cross-section characteristics^27^ and spatial diversity. In the second step, we established an optimization mathematical model for air-based radar with separated transmission and reception. In the third step, we optimized and solved the formation configuration of “1 transmit, n receive” cooperative detection for air-based radar with separated transmission and reception using the improved Artificial Fish Swarm Algorithm. We described the specific steps in detail. Finally, we used our self-developed software to perform simulation validation analysis, which successfully verified the effectiveness of our proposed method. We hope that the results of this study will inspire other scientists and lead to more research on rewriting the rules of air combat.

Multi-platform collaborative detection provides a solid foundation for the theory of air-based radar networking and paves the way for further development of multi-platform collaborative missions. In summary, the contributions of this paper are as follows:

1. In the field of radar networking, we have leveraged knowledge from both land-based and ship-based radar systems to implement the concept of multi-site radar in aerial combat. Moreover, we use a distributed radar networking architecture to maximize the safety of our own aircraft while laying the groundwork for future collaborative tactical strikes.
2. The efficiency of the improved Artificial Fish Swarm Algorithm is significantly higher than that of other relevant algorithms. While ensuring efficiency, the effectiveness of this method remains relatively high among similar algorithms.
3. This method has clear logic and strong interpretability. Its fast convergence speed in single-cycle operation can meet the real-time requirements of aerial combat. This method can be used stably even in situations with incomplete enemy information, which is essential in military applications.
4. The collaborative formation method proposed in this paper can be unified and commanded through ground stations or airborne command aircraft, enabling real-time sharing of the entire battlefield situation information. After completing comprehensive processing and situational assessment analysis of the battlefield information, the command platform can rapidly and scientifically develop combat plans based on task requirements, battlefield situation, and available resources, and send them to launchers and receivers to guide their completion of tasks such as situational awareness, target strike, and damage assessment. This approach has strong guiding significance for improving military capabilities.

## Preliminaries

### Background hypothesis

Based on reference^28^, we can formulate the following hypotheses: The operational scenario entails an initial separation distance of 300 km between friendly and hostile forces. The friendly mission involves utilizing coordinated tactics among its aircraft to conduct detection while dynamically adjusting its formation in real-time to maximize detection efficiency. The friendly forces also remain prepared to engage in tactical strikes against enemy aircraft at a moment's notice. Conversely, the enemy mission involves detecting our friendly forces' launch radar upon activation and dispatching combat aircraft for coordinated strikes, assuming the enemy's early warning aircraft can detect our launch radar. However, the enemy combat aircraft has yet to enter the detection range of our launch aircraft. To rapidly engage our friendly aircraft, the enemy aircraft will proceed in a direct line towards our launch aircraft, preparing to fire missiles. The initial situation between the two parties is that of a head-on encounter.

### Detection principle

**Figure 1:**
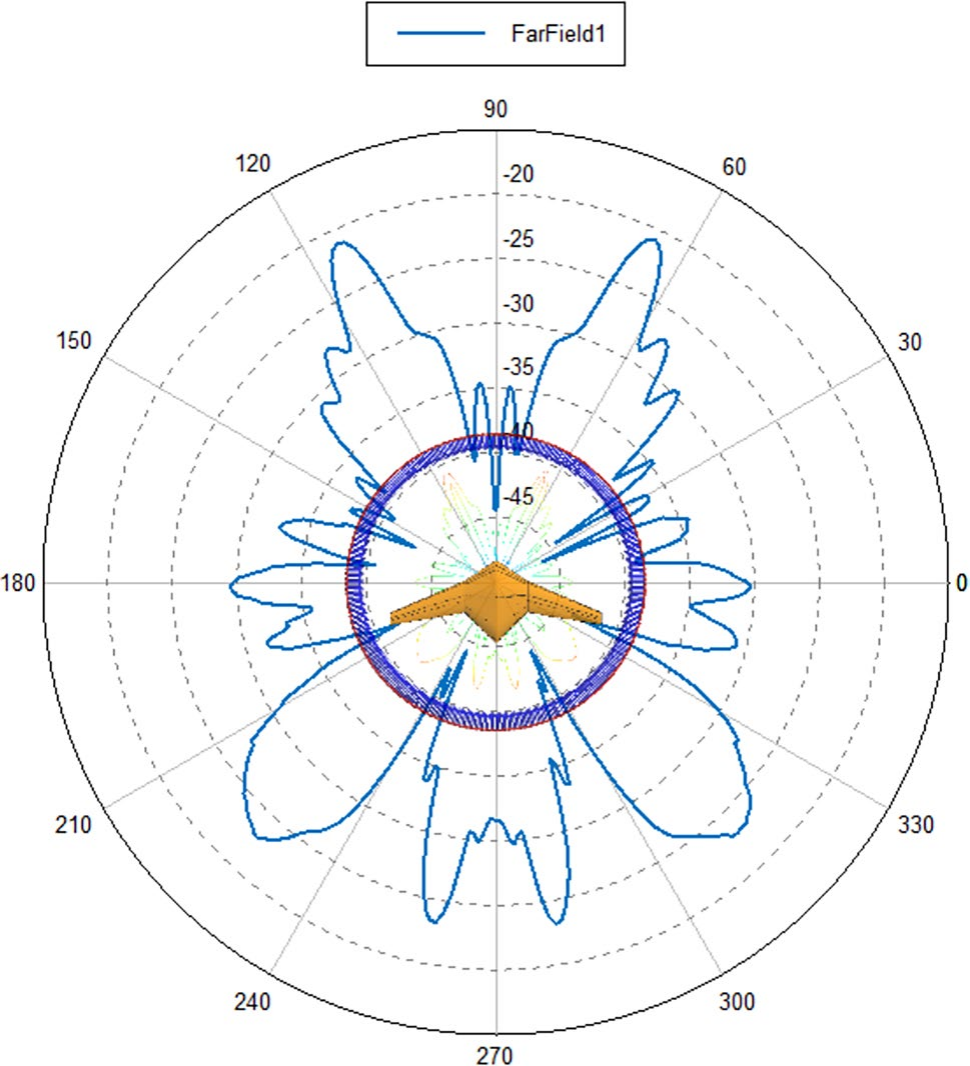
RCS simulation of a certain type of UAV.

**Figure 2:**
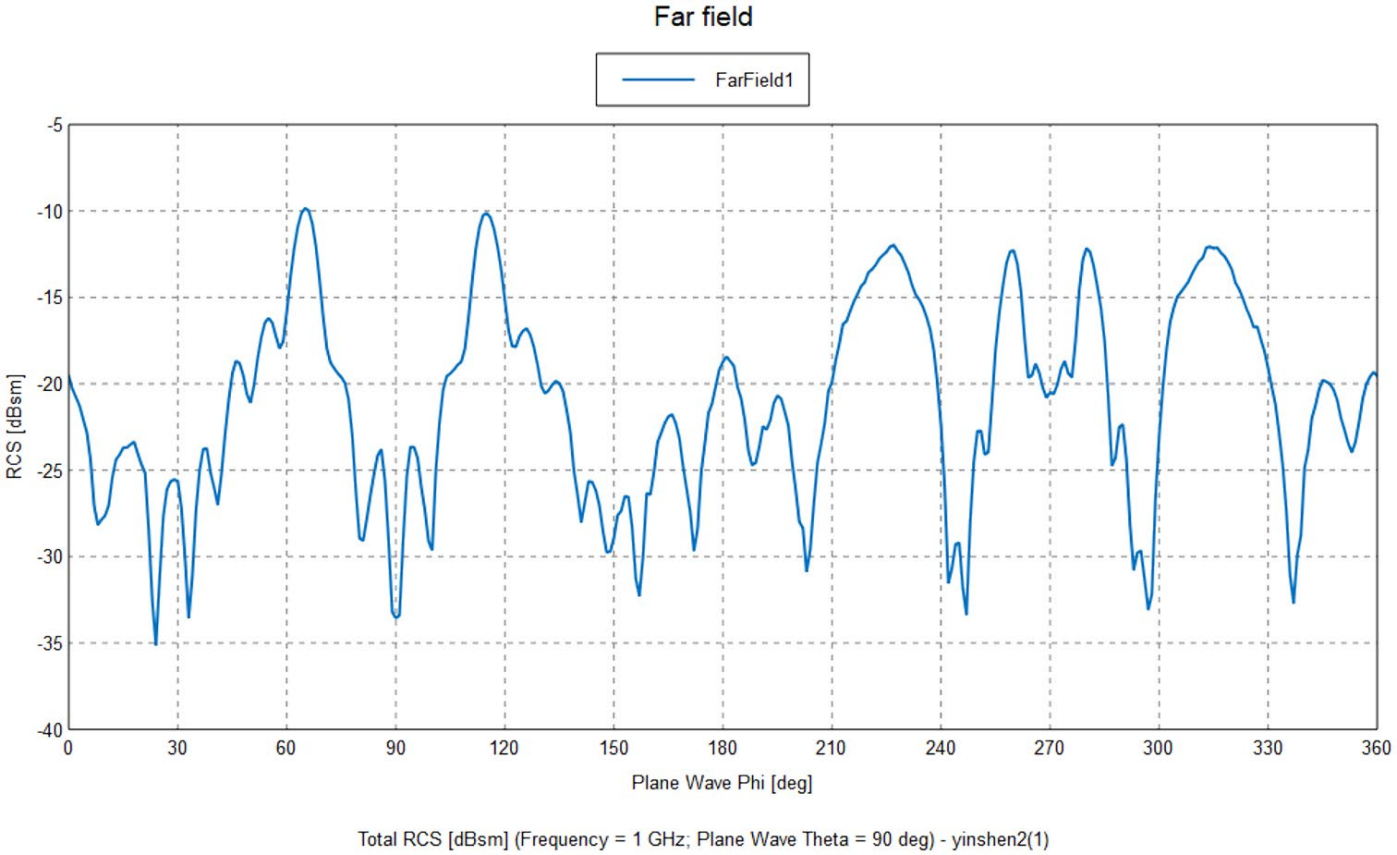
Schematic diagram of peak RCS curve of a certain type of UAV.

The shape and flight attitude of fighter aircraft significantly impact their Radar Cross Section (RCS). The concept of space diversity is applied in the air-based radar network to enhance the reliability and stability of target detection and tracking^29^. This is achieved by using multiple receiving stations to receive radar signals. In this study, a particular model of unmanned aerial vehicle was selected for RCS simulation. In this study, a specific model of unmanned aerial vehicle (UAV) was selected, and RCS (Radar Cross Section) simulations were conducted using the FEKO software. The RCS simulation results are presented in Fig. 1, indicating significant differences in RCS when the radar wave illuminates the target from different directions. As demonstrated in Fig. 2, RCS is relatively high in certain angular domains. Effective avoidance of directions with significantly reduced RCS characteristics of the enemy target by the detection device and detection from directions where target RCS values are higher would likely lead to successful target detection at the original range.

The effectiveness of target detection using single-station and multi-station detection differs significantly from that of single-unit detection. Research and simulation experiments have demonstrated^30^ that the radar cross-section (RCS) of fighter jets exhibits a greater forward and lateral scattering and a smaller backward scattering^31^. When a single-station radar detects a target, the distance of exposure in the direction of the target's nose cone is short, while the distances of exposure in the lateral and rear directions are relatively large, but the radar signal's peak is narrow, making it difficult for the radar to capture the echo signal. In contrast, when using multi-station detection, the exposure distances in the lateral and rear directions of the target are relatively large, making it easier for the radar to capture the echo signal. Therefore, it is evident that single-station radar has significant limitations in detecting airborne targets, while the air-based separated transmit/receive radar network has great potential in target detection.

**Figure 3:**
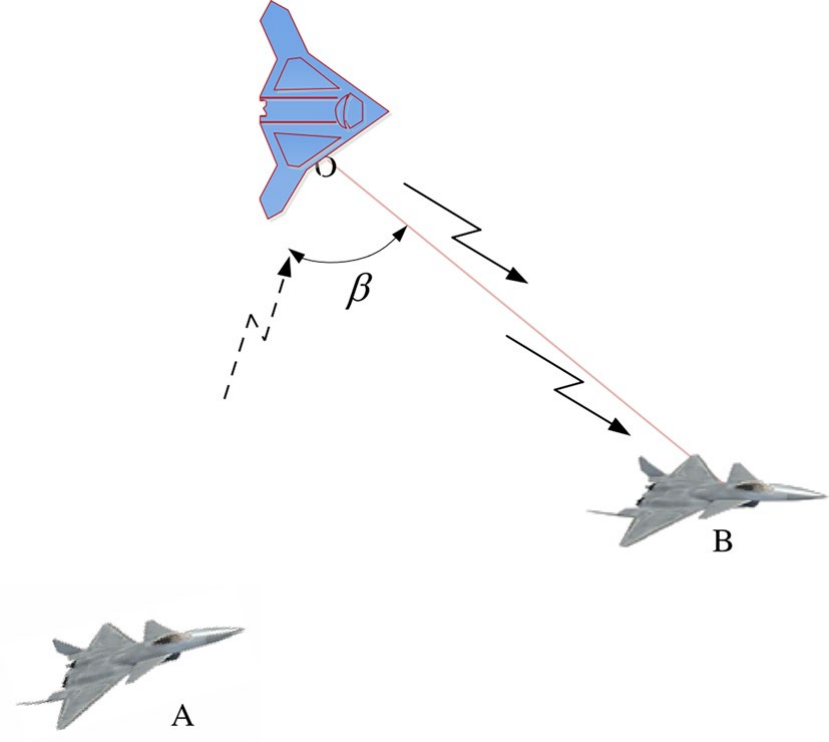
Illustration of the dual-site radar detection formed by the collaborative detection of two radars through dual-machine coordination.

In summary, the Radar Cross Section (RCS) of a target varies with different postures and polarization methods, which has a decisive impact on the detection efficiency of the airborne radar network. As depicted in Fig. 3, this characteristic is utilized by transmitting radar signals from a transmitter and receiving them from two or more receivers to achieve multi-angle detection and full coverage of the target^32^. This not only enables the reception of radar echoes in specific directions but also allows each receiving node's radar to remain electromagnetically silent, ensuring the preservation of its own security. This approach achieves the purpose of tracking and detecting targets over a large area and long distances, while consuming fewer combat resources in any operational environment and target state.

**Figure 4:**
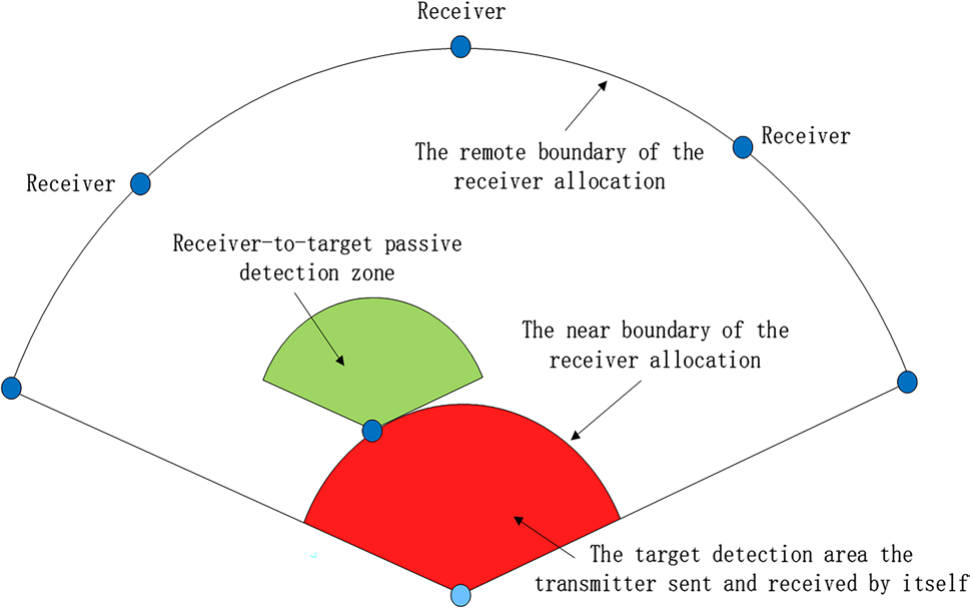
Schematic diagram of Space—based radar network transceiver—separate cooperative detection configuration.

Figure 4 depicts a Space—based radar network transceiver—separate cooperative detection configuration. The configuration includes nodes represented by light blue and dark blue colors, which respectively denote transmitters and receivers. The transmitters are equipped with active detection radar, while the receivers are equipped with passive detection radar, and are configured with near-field and far-field distances, respectively representing the configurations of the receivers in silent mode. Each receiver and transmitter can constitute an independent airborne radar station. During mission execution, the preceding receiver radar remains in a silent reception mode while the subsequent transmitter radar remains intermittently switched on for transmission, enabling the air-to-ground radar network to detect targets efficiently in the detection area with the farthest possible range and the smallest number of participating platforms while maintaining its survivability. This approach also meets the requirements for self-frequency control of the transmitter and receiver, reduce the probability of discovery and improve the survival ability of our own side.

## Methodology

**Figure 5:**
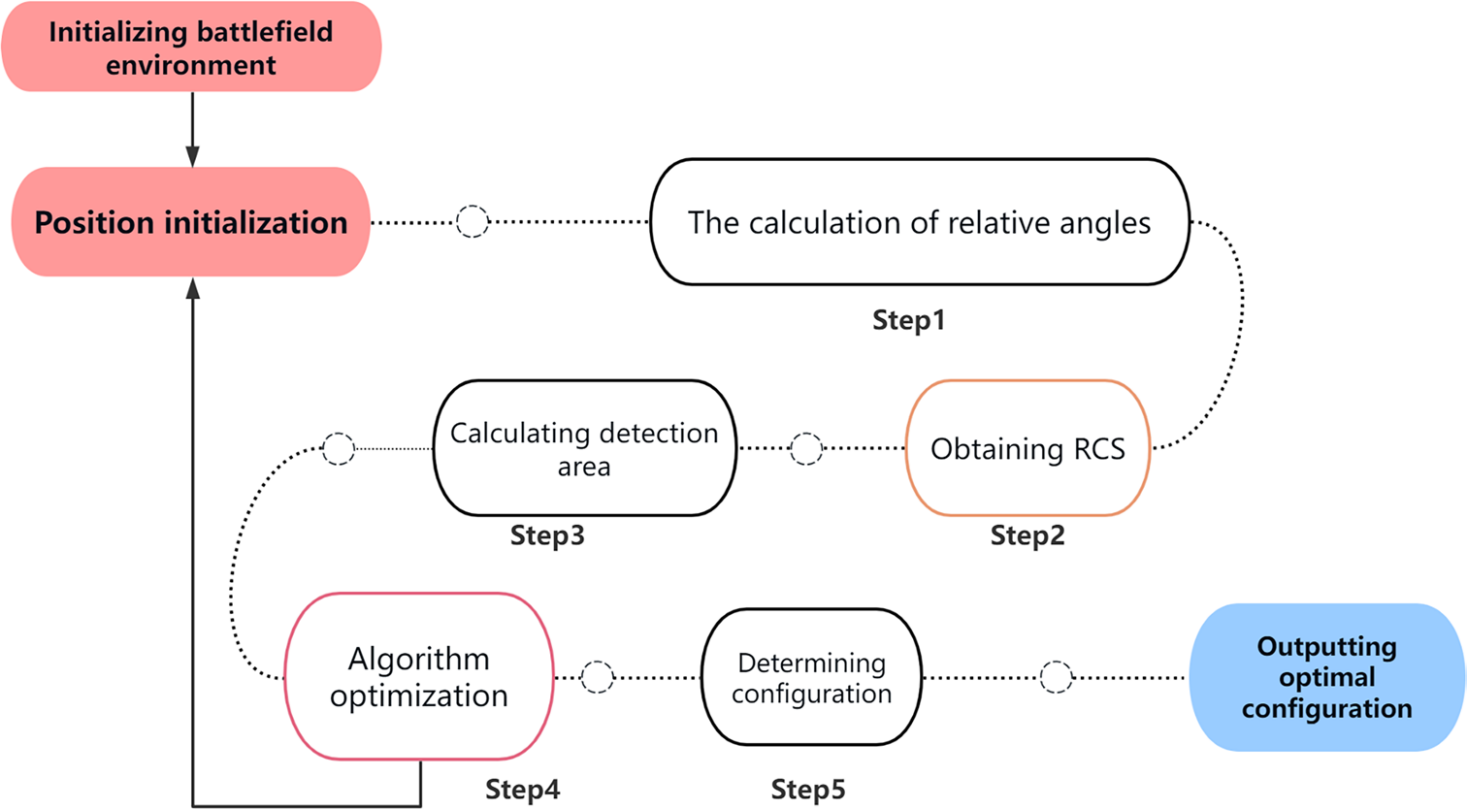
The entire process flowchart.

The entire process is illustrated in Fig. 5. In this chapter, we first establish a model for swarm collaboration in anti-stealth airspace coverage, and propose an improved method for solving the airspace coverage problem. Next, we define four evaluation indicators and propose an effective method for calculating the detection area, which is used to obtain an optimized objective function through a weighted sum. Finally, based on the study of the one-to-many configuration, we optimize the 1TnR spatial configuration using an improved Artificial Fish Swarm Algorithm.

### Construction of the optimization model

1. Evaluation model.

The scattering characteristics of a multiple-site radar system can be simplified for analysis purposes by considering the scattering characteristics of a dual-site radar system. When accounting for the propagation and attenuation factors of a dual-site radar system, the dual-site radar equation can be expressed as^33,34^:

1 $$ (R_{T} R_{R} )_{\max }^{2} = \frac{{P_{T} G_{T} G_{R} \lambda^{2} \sigma_{B} F_{T}^{2} F_{R}^{2} }}{{(4\pi )^{3} P_{R\min } }} $$

Within the given equations, $R_{T}$ represents the distance between the transmitter and the hostile aircraft, The parameter $R_{R}$ denotes the distance between the receiver and the enemy aircraft.; $P_{T}$ represents the power of the transmitter, The capacity of dual-station radar to detect enemy aircraft is characterized by the parameter $P_{R}$; The variables $G_{T}$ and $G_{R}$ represent the antenna gain of the transmitter and receiver, respectively, in the context of wireless communication; The wavelength at which radar operates is $\lambda$; $F_{T}$、$F_{R}$ are directional propagation factors for wireless communication, representing the transmission and reception patterns respectively; The parameter $\sigma_{B}$ refers to the radar cross-section of the hostile aircraft as detected by the dual-station radar system, and is denoted by $\sigma_{B} = \sigma (\alpha_{T} ,\beta_{T} {;}\;\alpha_{R} ,\beta_{R} )$, In this expression, $\alpha_{T}$ and $\beta_{T}$ represent the azimuth and elevation angles, respectively, of the enemy aircraft targeting the transmitter and receiver. Similarly, $\alpha_{R}$ and $\beta_{R}$ represent the azimuth and elevation angles, respectively, of the enemy aircraft targeting the receiver and transmitter. To ensure clarity, azimuth angle refers to the horizontal angle measured clockwise from true north, and elevation angle refers to the vertical angle measured from the horizontal plane. Given the known parameters, $\sigma_{B}$ (RCS) of an enemy aircraft's dual-station radar can be determined by accessing a dual-station RCS database.

Furthermore, given other known parameters, the dual-station radar equation can be employed to calculate the detecting power of the enemy aircraft $P_{R}$;

The evaluation metrics that affect the detection efficiency of bistatic radar systems include the defensive coverage area $S_{F}$, the maximum distances from the transmitter and receiver to the detection boundary $T_{far}$、$R_{far}$, and the maximum width of the detection area $W_{\max }$.

The stated defense area, denoted as $S_{F}$, can be expressed as the summation of the radar detection area $S_{d}$, and the protection area $S_{p}$, that is:

2 $$ S_{F} = S_{d} + S_{p} $$

3 $$ S_{d} = \sum\limits_{{{\text{i}} = 1}}^{{\text{n}}} {S_{i}^{d} } - \sum\limits_{{{\text{i}} = 2}}^{{\text{n}}} {S_{i}^{d\Omega } } $$

4 $$ S_{p} = \sum\limits_{{{\text{i}} = 1}}^{{\text{n}}} {S_{i}^{p} } - \sum\limits_{{{\text{i}} = 2}}^{{\text{n}}} {S_{i}^{p\Omega } } $$

In this context, the variable $S_{d}$ refers to the total area of the effective detection zones of the radar system, which is obtained by subtracting the overlapping regions from the sum of all the detection areas. Meanwhile, the variable $S_{p}$ represents the area of the protection zone, where any target entering the zone can be detected and tracked by the radar system as soon as it crosses the inner boundary. Let $n$ be the total number of receivers, and $i$ be the index of a particular receiver. We define $S_{i}^{d}$ as the area of the detection region for the $i$ receiver, and $S_{i}^{d\Omega }$ as the sum of overlap areas between the detection region of the $i$ receiver and those of receivers with indices smaller than $i$. Similarly, we define $S_{i}^{p}$ as the area of the defense airspace formed by the line connecting the rear boundary of the detection region for the $i$ receiver and the transmitter, and $S_{i}^{p\Omega }$ as the sum of overlap areas between the defense airspace of the $i$ receiver and those of receivers with indices smaller than $i$.

Based on four performance evaluation indicators that impact the effectiveness of dual-site radar detection, we have developed an assessment model:

5 $$ \begin{aligned} F & = w_{1} S_{F} /S_{a} + w_{2} T_{far} /L_{a} + w_{3} R_{far} /L_{a} \\ & \;\;\; + w_{4} W_{\max } /(X_{\max } - X_{\min } ) \\ \end{aligned} $$

6 $$ S_{a} = (X_{\max } - X_{\min } )(Y_{\max } - Y_{\min } ) $$

7 $$ L_{a} = \left( {(X_{\max } - X_{\min } )^{2} + (Y_{\max } - Y_{\min } )^{2} } \right)^{1/2} $$

In this study, we propose a metric, denoted by $F$, to evaluate the effectiveness of optimizing the configuration of our multi-aircraft cooperative detection formation with separate transmission and reception systems. The maximum distances from the transmitter and receiver to the detection boundary are denoted by $T_{far}$ and $R_{far}$, respectively, while $W_{\max }$ represents the maximum width of the detection area. The concept of $S_{a}$ refers to the detection area of our transmitter and receiver, and $L_{a}$ denotes the diagonal length of the detection area. The coordinates of the detection area on the X and Y axes are represented by ${ [}X_{\min } ,X_{\max } {] }$ and ${[}Y_{\min } ,Y_{\max } {]}$, respectively. The weights $\omega_{1}$, $\omega_{2}$, $\omega_{3}$ and $\omega_{4}$ correspond to the evaluation criteria of detection efficiency, namely, $S_{F}$、$T_{far}$、$R_{far}$ and $W_{\max }$, respectively, indicating the relative importance of each criterion and satisfying the condition $\sum\limits_{i = 1}^{4} {\omega_{i} } = 1$.

2. Calculation of the detection area.

This paper uses spatial partitioning method to solve the detection area. Next, the calculation process of the effective detection area will be detailed according to the steps.

***Step1:*** To initialize the surveillance airspace.

Assume that the airspace under surveillance is denoted by $\Omega_{s}$. The maximum and minimum values along the x-axis are represented by $X_{\max }$ and $X_{\min }$, respectively, while the maximum and minimum values along the y-axis are represented by $Y_{\max }$ and $Y_{\min }$. The surveillance airspace is divided into a spatial grid using the concept of spatial partitioning, with each grid having a minimum unit of $\Delta {\text{x}} \times \Delta {\text{y}}$. Here, $\Delta {\text{x , }}\Delta y$ denote the length and width of the grid unit, respectively, and are generally assumed to be equal, resulting in square-shaped grids at every height level. The area of each small square after grid partitioning is $\Delta S = \Delta {\text{x}} \times \Delta {\text{y}}$. Within the surveillance area, each grid unit is considered as a hypothetical enemy target point. The central coordinate of any target point can be represented as ${(}X_{\min } + i_{x} \Delta {\text{x}} + \Delta {\text{x}}/2,Y_{\min } + i_{y} \Delta y + \Delta y/2)$, where 0 ≤ $i_{x}$ < $K_{x}$, 0 ≤ $i_{y}$ < $K_{y}$。The grid size can be adjusted by changing the values of $\Delta {\text{x}}$ and $\Delta {\text{y}}$. For this scenario, the detection airspace parameter is set to $\Omega_{S} = [ - 300,300]{\text{km}}\; \times \;[0,\;300]\;{\text{km}}$, and the grid unit step length is set to $\Delta {\text{x = }}\Delta {\text{y = 2}}\;{\text{km}}$.

***Step2:*** Determine whether the imaginary enemy target point $Q_{k} (x_{k} ,y_{k} )$ is located within the scanning Angle of the transmitter and receiver.

Let $\theta_{\max }$ denote the maximum angle of the radar wave between the transmitter and receiver. If $\arctan \frac{{x_{k} }}{{y_{k} }} \le \frac{{\theta_{\max } }}{2}$, $\arctan \left| {\frac{{x_{k} - x_{Ri} }}{{y_{k} - y_{Ri} }}} \right| \le \frac{{\theta_{\max } }}{2}$, and $y_{k} > y_{Ri} \ge 0$, the target location can proceed with the subsequent steps. Otherwise, the point shall be skipped, and the assessment shall continue with the next location.

***Step3:*** Determine whether the target point can be detected.

By utilizing the hypothetical coordinates of the target point $Q_{k} (x_{k} ,y_{k} )$, the relative azimuth and elevation angles with respect to the transmitting and receiving stations can be computed. Through querying the dual-station RCS database of the target, the corresponding RCS value $\sigma_{B}$ can be obtained. The distance from the target point to the transmitting and receiving stations of the dual-base radar is calculated, and this distance is then inserted into the following equation to derive the detection power of the dual-station radar for the enemy aircraft located within the grid unit.

8 $$ P_{k} = \frac{{P_{T} G_{T} G_{R} \lambda^{2} \sigma_{B} F_{T}^{2} F_{R}^{2} }}{{(4\pi )^{3} (x_{k}^{2} + y_{k}^{2} )\left[ {(x_{k} - x_{Ri} )^{2} + (y_{k} - y_{Ri} )^{2} } \right]}} $$

When $P_{k} \ge P_{R\min }$, the detection system implemented by our team is deemed capable of identifying enemy aircraft present at the target point, leading to an overall increase in the coverage area: $\Delta x \times \Delta y$. Conversely, the detection system is unable to recognize aircraft within the corresponding grid cell, thereby causing no change in the coverage area. The algorithm then proceeds to determine whether all grid cells have been exhaustively searched. If this condition is satisfied, the computation is terminated; otherwise, the next unexplored grid cell is selected, and the above steps are repeated.

After surveying all target points within the designated alert areas, it is possible to determine whether each small square can be detected. The summation of all detectable areas yields the detection range of the dual-baseline radar system for the target region. It is worth noting that the solution obtained through this algorithm is only an approximation, as the precision of the approximation increases as the spatial discretization size △x, △y decreases, but at the cost of increased computational complexity.

The above steps provide a detailed description of the calculation method for the effective detection area. In addition to the effective detection area, the calculation of the protective area is also necessary. If we consider the entire detection area of the dual-machine collaborative detection as a virtual “macro radar” detection area, and the enemy aircraft is detected and tracked after passing through the receiver’s detection area due to the forward movement of the aircraft cluster. Therefore, the airspace formed by the rear boundary of the receiver’s detection area and the line connecting the transmitter can be regarded as the effective protection area of the aircraft cluster. By utilizing the boundary of the detection area and the scanning angle of the radar space, the area of the protective area can be calculated as follows: the area formed by connecting the rear boundary of the receiver’s detection area to the transmitter is defined as the protective area.

3. Summary

The problem addressed in this paper is ultimately summarized as an optimization problem:

Specifically, determining the optimal relative positions of a transmitter and multiple receivers in a one-to-many scenario, where a single transmitter corresponds to multiple receivers.

9 $$ Best(S_{1} ,R_{1} ,R_{2} \ldots R_{n} ),\;{\text{inside}}\left\{ \begin{gathered} S_{1} = (x_{1} ,y_{1} ) \, \hfill \\ R_{1} = (x_{2} ,y_{2} ) \hfill \\ R_{2} = (x_{3} ,y_{3} ) \hfill \\ \, \cdots \hfill \\ R_{n} = (x_{n} ,y_{n} ) \hfill \\ \end{gathered} \right., $$

The coordinates of the transmitter at the optimal configuration are denoted by $S_{1}$, while the coordinates of the receiver relative to the transmitter at the optimal configuration are denoted by $R_{1} ,R_{2} \ldots R_{n}$.

The aforementioned coordinates are subject to the following set of constraints:

10 $$ \left\{ \begin{gathered} R_{near} < \sqrt {(x_{2} - x_{1} )^{2} + (y_{2} - y_{1} )^{2} } < R_{far} \hfill \\ R_{near} < \sqrt {(x_{3} - x_{1} )^{2} + (y_{3} - y_{1} )^{2} } < R_{far} \hfill \\ \, \cdots \hfill \\ R_{near} < \sqrt {(x_{n} - x_{1} )^{2} + (y_{n} - y_{1} )^{2} } < R_{far} \hfill \\ 30^{ \circ } < \arctan \frac{{y_{2} - y_{1} }}{{x_{2} - x_{1} }} < 150^{ \circ } \hfill \\ 30^{ \circ } < \arctan \frac{{y_{3} - y_{1} }}{{x_{3} - x_{1} }} < 150^{ \circ } \hfill \\ \, \cdots \hfill \\ 30^{ \circ } < \arctan \frac{{y_{n} - y_{1} }}{{x_{n} - x_{1} }} < 150^{ \circ } \hfill \\ \end{gathered} \right. $$

The terms $R_{near}$ and $R_{far}$ are used to respectively denote the near-field distance and the far-field distance.

### Solution based on the improved ASFA

From the perspective of aircraft swarm coordinated control methods, the problem of swarm coordinated search can be transformed into an optimization problem by designing reasonable algorithms. This approach takes into consideration the autonomy, intelligence, and robustness of biological swarm systems, as well as their similarities to unmanned aerial vehicle (UAV) swarm systems. Researchers have attempted to solve the key issues and technical bottlenecks in the coordination, decision-making, and control of UAV swarms by studying the intrinsic mechanisms underlying coordinated and coherent motion and decision-making in groups such as flocks of birds, schools of fish, and colonies of bees.

In nature, individuals in biological swarms only follow simple behavioral rules and interact with their neighboring peers and the environment, yet they can adaptively adjust their behavioral states and patterns. Despite the limited individual intelligence and behavioral capabilities, driven by the behavioral and decision-making mechanisms within the swarm, the entire swarm can exhibit complex intelligent behaviors from local to global scales. They can accomplish complex team activities such as migration, habitation, foraging, nest building, and defense, presenting a coordinated and organized self-organizing state of the swarm.

**Figure 6:**
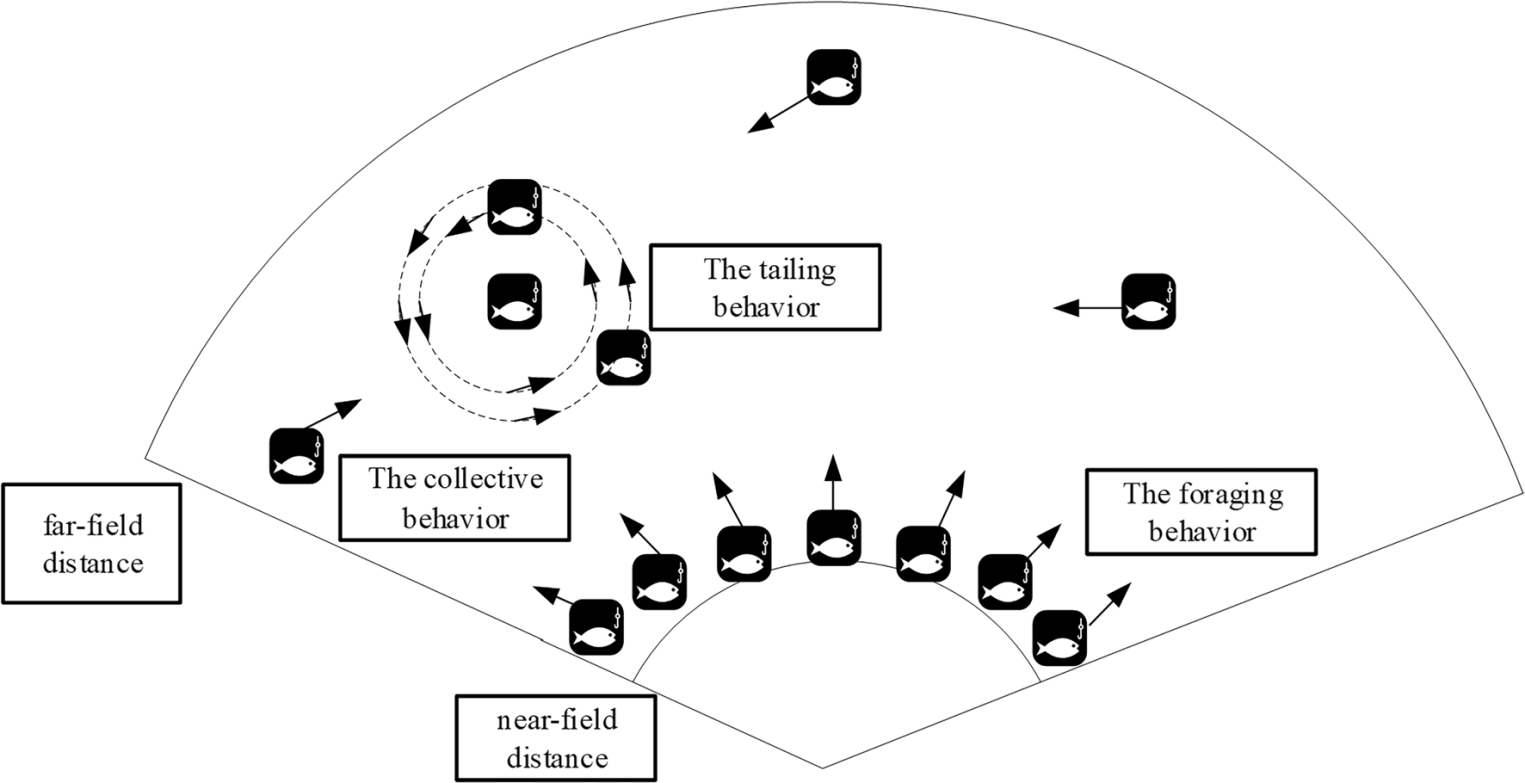
Improved Artificial Fish Swarm Algorithm behavior diagram.

The Artificial Fish Swarm Algorithm (AFSA) is an intelligent optimization algorithm developed based on the study of natural fish behavior. It is designed to solve optimization problems by simulating the behavior of fish in a water environment. In this algorithm, fish are capable of independently or collectively searching for areas with abundant nutrients by either following other fish or exhibiting foraging behavior. By comparing the fitness values of fish after clustering, tail-chasing or foraging, the AFSA algorithm is able to determine the solution to the optimization problem. However, the initialization of the AFSA algorithm requires fixed settings of parameters such as the visual range and step size of the artificial fish swarm, which can affect the efficiency of obtaining the optimal solution and the computational complexity of the algorithm. Therefore, this study proposes and implements an improved AFSA-based distributed cooperative detection configuration calculation method for aircraft swarm using the characteristics and internal mechanisms of fish foraging behavior. This method aims to solve the problem of multi-aircraft cooperative target search. The proposed method leverages the advantages of combat aircraft and efficiently achieves rapid target search in unknown spatial locations in the given task scenario, effectively improving the cooperative detection capability of the aircraft swarm. The schematic of the improved AFSA algorithm is shown in Fig. 6.

**Figure 7:**
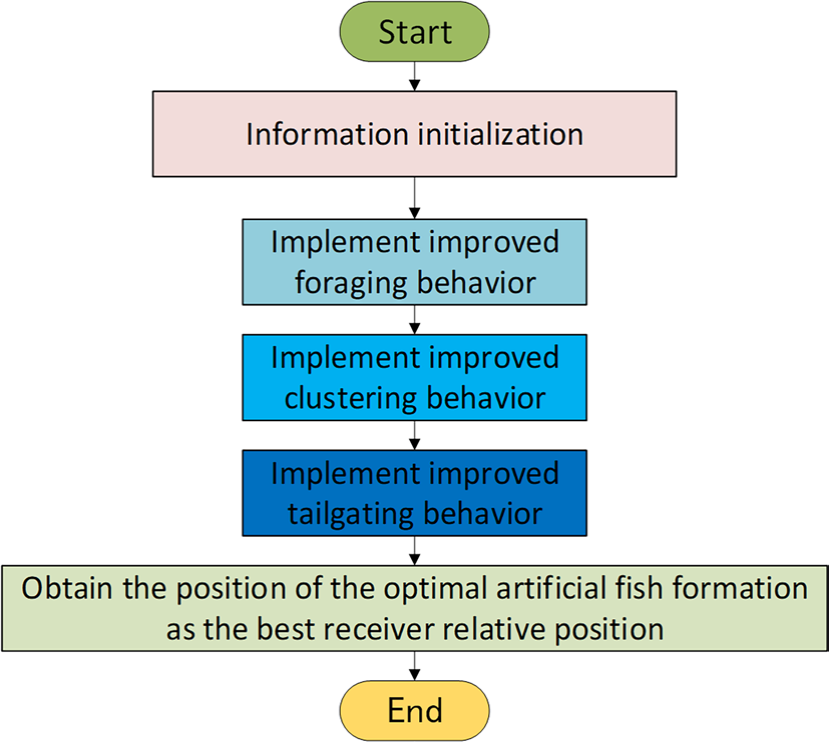
The overall flow chart of the improved Artificial Fish Swarm Algorithm.

The specific solution process is shown in Fig. 7:

***Step 1***. Initializes the deployment area of the receiver;

The deployment area of the receiver relative to the transmitter can be partitioned into n sections based on the number of receivers (n). Starting from the transmitter location as the origin of the ray emission, the space between the near and far boundaries of the transmitter detection is divided into n equal parts. Each part is considered a search area for one receiver, designated from left to right as region 1 through region n.

***Step 2***. Initialize the artificial fish swarm;

Considering the maximization of defense coverage area, it is necessary to ensure symmetry in the configuration of the receivers on both sides of the detection airspace bisector of the transmitter. Configuration optimization only needs to be performed on one side, while the other side remains symmetrical. Therefore, the initialization of the artificial fish swarm is only performed within the search area of receiver positions located on and to the left of the detection airspace bisector of the transmitter. For ease of description, the detection area of the receiver placed at the artificial fish position is defined as the artificial fish's detection area. This process includes the following steps:

***Step 2.1***. When the number of receivers is singular, artificial fish swarm initialization is carried out through the following two steps:

- To initialize an artificial fish swarm within the search area of the receiver position located on the perpendicular bisector of the transmitter detection space, the following steps were taken: firstly, a single artificial fish was generated at the intersection of the perpendicular bisector and the near-field boundary of the transmitter detection space. Subsequently, new artificial fish were generated at intervals of $\Delta d$ along the opposite direction of the line connecting the artificial fish and the transmitter. The position information of each generated artificial fish and its corresponding detection area were stored. The generation process was terminated once the distance between the newly generated artificial fish and the transmitter exceeded the range $R_{far}$.
- For the initialization of artificial fish schools in the remaining regions,$k$ artificial fish individuals with labels are generated on the boundary of each region using an equidistant distance partitioning method.

**Figure 8:**
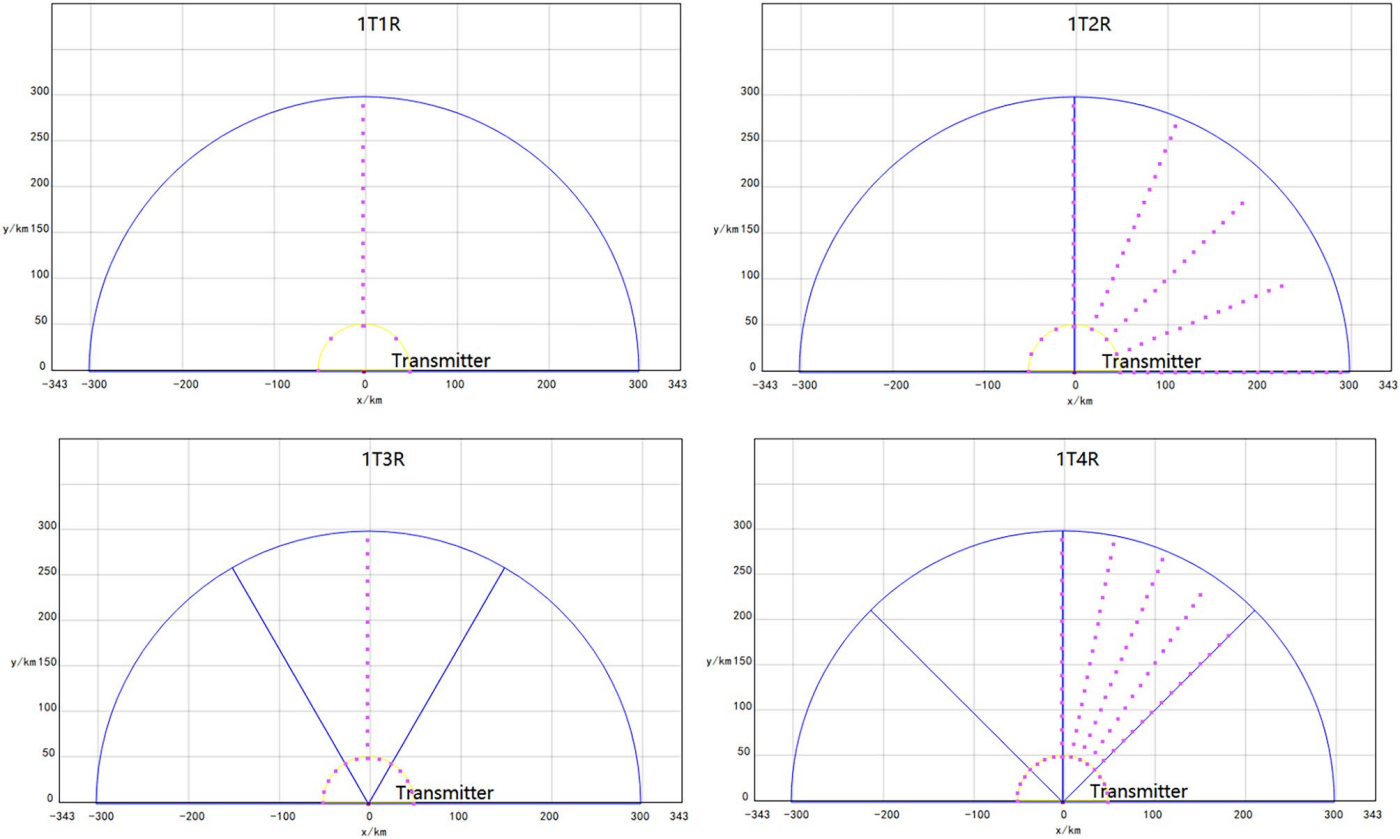
Schematic diagram of artificial fish school initialization.

***Step 2.2***. When the number of receivers is even, the initialization of the artificial fish swarm proceeds in two steps, as follows:

1. To initialize an artificial fish swarm in the search area to the left of the spatial bisector line of the transmitter detection space, the first receiver position is selected. Firstly, $k$ tagged individuals of the artificial fish are initialized on the near boundary line according to distance equalization. Next, for everyone, new artificial fish are generated forward at intervals of $\Delta d$ along the opposite direction of the line connecting the fish to the transmitter. If the newly generated artificial fish covers the spatial bisector line of the transmitter detection space, the position information of the fish and its detection area are stored; Otherwise, the fish is not generated. Generation ceases when the distance between the newly generated fish and the transmitter exceeds $R_{far}$.
2. Initialization of the artificial fish population in the remaining regions;K artificial fish individuals with labels are generated on the near boundary of each region using equidistant distribution based on distance;

The initialization process for the artificial fish is illustrated in Fig. 8.

**Figure 9:**
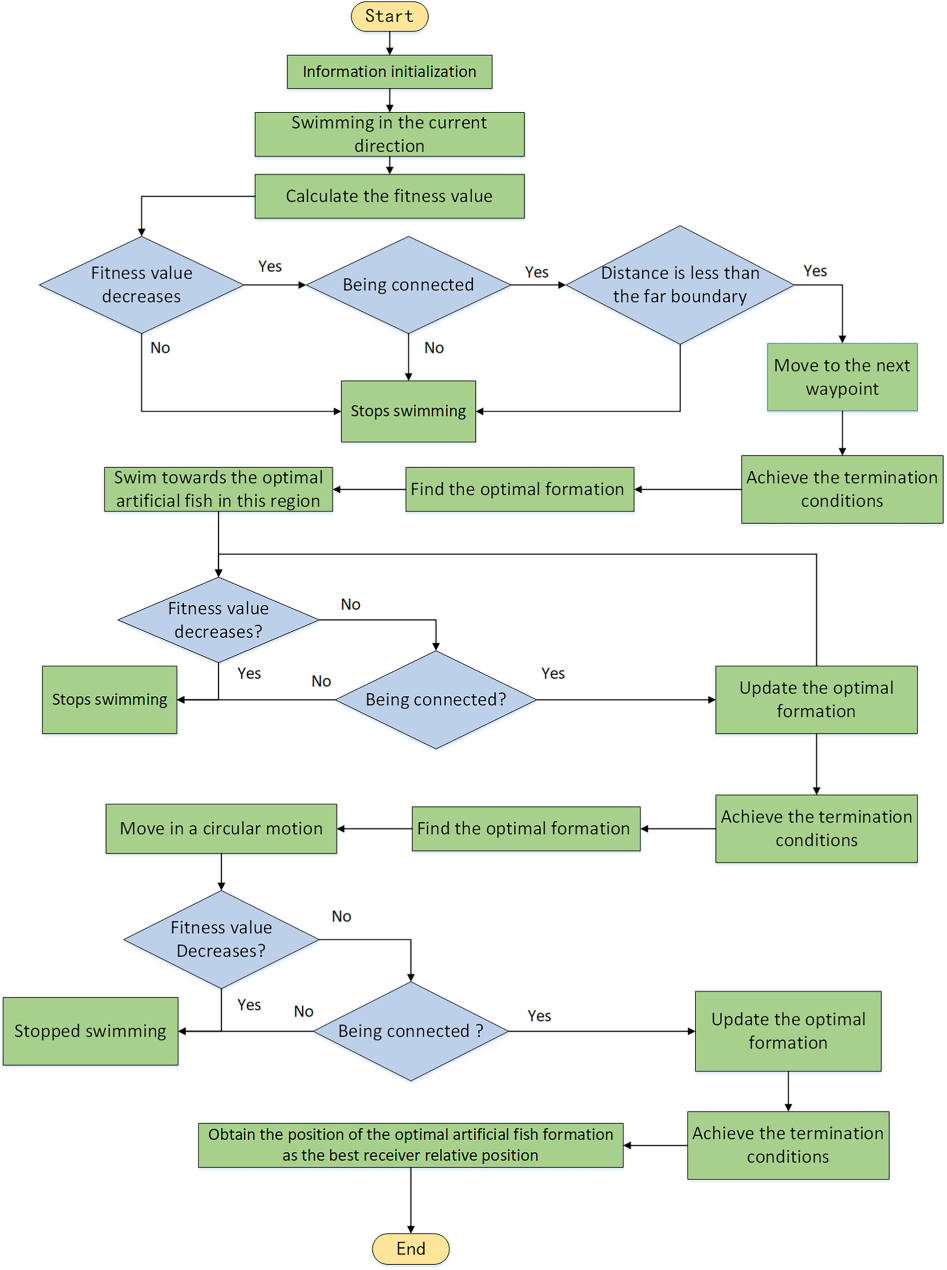
Process of IAFS.

***Step 3***. The foraging behavior of an artificial fish swarm is demonstrated through a specific process as depicted in Fig. 9.

***Step 3.1***. The current field of vision and step size of the artificial fish within region $p,\;p < n/2$ are adaptively adjusted.

$A_{pq}$ is defined as the q-th artificial fish searching for the receiver's position within the p-th region. The specific approach for this step is as follows:

Each artificial fish $A_{pq}$, $1 \le p \le n$, $1 \le q \le k$ calculates the average distance $D_{pq}^{t}$ between itself and $k - 1$ other fishes within the same region. The calculated average distance is used as the field of vision for the t-th iteration of the fish.

The step size $s_{pq}^{t}$ is then computed according to the following formula:

11 $$ D_{pq}^{t} { = }\frac{1}{k - 1}\sum\limits_{g = 1}^{k} {d_{pqg}^{t} } $$

12 $$ d_{pqg}^{t} = \left| {\left. {X_{pq}^{t} - X_{pg}^{t} } \right|} \right. $$

13 $$ s_{pq}^{t} = a \times D_{pq}^{t} $$

During the t-th iteration, the artificial fish $A_{pq}$ is utilized to search for the position of the p-th receiver. The distance between the artificial fish $A_{pq}$ and $A_{pg}$ is denoted by $d_{pqg}^{t}$. Furthermore, $X_{pq}^{t}$ and $X_{pg}^{t}$ represent the current positions of $A_{pq}$ and $A_{pg}$, respectively. The variable "$a$" signifies the step size coefficient,${0 < }a < 1$.

***Step 3.2***. To optimize the positioning of artificial fish within region $p \, p \le [n/2]$.

***Step 3.2.1***. Sequentially evaluating $k$ artificial fish within region $p$ from $p = n/2$ to $p = 1$, if the detection area of an artificial fish in the right adjacent area is connected to its own detection area, then steps 3.2.2–3.2.4 are executed sequentially. Otherwise, step 3.2.5 is executed.

***Step 3.2.2***. Based on the evaluation model of collaborative detection formation with multi-aircraft in separate sending and receiving roles, we calculate the optimization indicator values of formation configuration for each pair of artificial fish that form a connected detection area in the current region and its adjacent region to the right. We then identify the artificial fish with the highest optimization indicator value as the optimal formation configuration.

***Step 3.2.3***. First, the artificial fish with the highest optimized index value in the local area is directed to move a distance equal to its step size in the direction opposite to the line connecting it with the transmitter. Subsequently, the maximum optimized index value is computed for the formation configuration resulting from the artificial fish's alignment with its neighboring artificial fish in the adjacent region to the right. If the optimized index value after the artificial fish's movement is greater than that before its movement, the process is repeated. Otherwise, the artificial fish returns to its original position and ceases to move.

***Step 3.2.4***. After the artificial fish in each region have stopped moving, the connectedness criterion of the detection region is followed to sequentially select an artificial fish from each region for permutation and combination. The optimization metric for each combination corresponding to the formation configuration is computed, and the artificial fish in the formation with the highest metric value are labeled as the optimal artificial fish.

***Step 3.2.5***. Retreat to Step 1, wherein the segmented region count is increased by one, followed by the re-division of the search area for the receiver's location.

***Step 4***. The collective behavior of artificial fish swarm is executed through a specific process, which is illustrated in Fig. 9.

***Step 4.1***. To adaptively adjust the current field of view and step size of suboptimal artificial fish.

***Step 4.2***. The algorithm proceeds from region $p = n/2$ to region $p = 1$, sequentially evaluating $k$ artificial fish in region $p$. If the distance between a non-optimal fish and the optimal fish within its region exceeds the prescribed step length and the former fish can swim to a position where its detection region is connected to that of another fish in the adjacent region, then the non-optimal fish moves towards the optimal fish at its maximum step size. Conversely, the fish stops moving if the above conditions are not met.

***Step 4.3***. Based on the principle of connectivity within the exploration regions, a systematic approach is employed to select individual artificial fish in each region for permutation and combination. Optimization criteria are then calculated for each combination to identify the configuration with the maximum value. The artificial fish within the optimal configuration are subsequently identified and marked as the optimal artificial fish for the given region.

***Step 4.4***. To determine whether all artificial fishes have ceased swimming, the procedure should proceed to Step 5 if affirmative, or alternatively, revert to executing Step 4.1 in the case of a negative result.

***Step 5***. The artificial fish swarm exhibits tailing behavior, and the detailed process is illustrated in Fig. 9.

***Step 5.1***. Adaptive adjustments should be made to the current field of vision and step size of non-optimal artificial fishes.

***Step 5.2***. Starting from $p = n/2$ and proceeding to $p = 1$ in sequence, $k$ artificial fishes in the region $p$ are evaluated. Following the completion of swarm behavior, artificial fishes that have stopped in each region orbit the optimal artificial fish in a circular motion. If the next position of a non-optimal artificial fish after circling the optimal artificial fish at a step length guarantees that its detection area is connected to the detection area of another artificial fish in the adjacent right region, then the non-optimal artificial fish moves one step in orbit around the optimal artificial fish. Otherwise, the non-optimal artificial fish ceases swimming.

***Step 5.3***. In order to determine whether all artificial fish are in a state of static suspension or have completed a 360-degree circular movement, the procedure outlined in Step 5.1 is executed. If the aforementioned condition is satisfied, Step 6 is carried out; otherwise, the system returns to Step 5.1 for further evaluation.

***Step 6***. To improve the search for the optimal formation configuration, the position of the optimal artificial fish formation obtained after performing three behaviors was selected as the optimal position for configuring our receiver, thus resulting in a successful search for the optimal formation configuration.

This paper presents an improved Artificial Fish Swarm Algorithm-based method for optimizing the formation configuration of an airborne radar receiver-transmitter detection team. The method optimizes the process of formation configuration search, addresses the problem of slow computation time in traversal algorithms, and improves upon traditional Artificial Fish Swarm Algorithms by reducing computational complexity and avoiding local optima, theoretically enhancing the detection range and efficiency for enemy targets.

## Experiments and analysis

To validate the effectiveness and practicality of the proposed algorithm, this study conducted various algorithm comparison experiments and analyses. Specifically, the brute-force traversal method, traditional particle swarm optimization algorithm, and improved Artificial Fish Swarm Algorithm were employed to optimize the formation of “one transmitter-one receiver,” “one transmitter-two receivers,” and “one transmitter-three receivers.” The effectiveness and practicality of the proposed algorithm were confirmed through analysis of the simulation results. Furthermore, it was demonstrated that in solving the optimization problem of spatial configuration, the improved Artificial Fish Swarm Algorithm outperforms the traversal method and particle swarm optimization algorithm in terms of both speed and stability of solutions.

The experimental setup was as follows: all algorithmic code was programmed in C++ language and executed on the QT platform. The physical platform consisted of multiple Dell desktop computers equipped with Intel Core i7-9750H 2.60 GHz processors, 8 GB of memory, and a 64-bit operating system.

### Experimental environment

This study is conducted under the auspices of Shenyang Aerospace University and the Key Laboratory of Advanced Flight Control and Simulation Technology in Liaoning Province. The research direction focuses on air combat decision-making and flight simulation.

**Figure 10:**
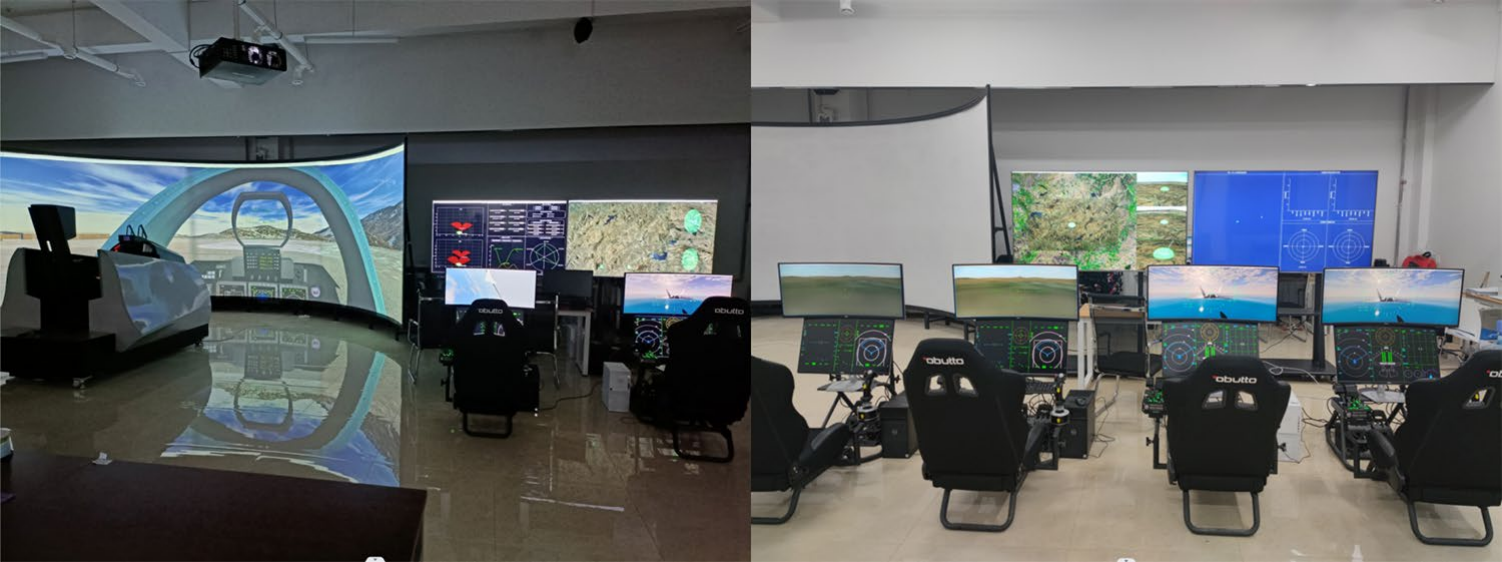
The experimental setup utilized in this study included a full-scale cockpit of a combat aircraft and a three-channel simulation testing platform.

In military applications, the system used is typically required to exhibit exceptional stability and high efficiency to ensure optimal performance of military equipment in actual combat scenarios. The main objective of this study is to design and improve optimization algorithms that not only reduce computation time for optimal configurations in multi-aircraft cooperative detection but also produce configurations with superior performance. This research lays the groundwork for future studies on multi-aircraft cooperative detection. Several experimental devices used in this study are illustrated in Fig. 10.

### Semi-physical simulation validation platform

**Figure 11:**
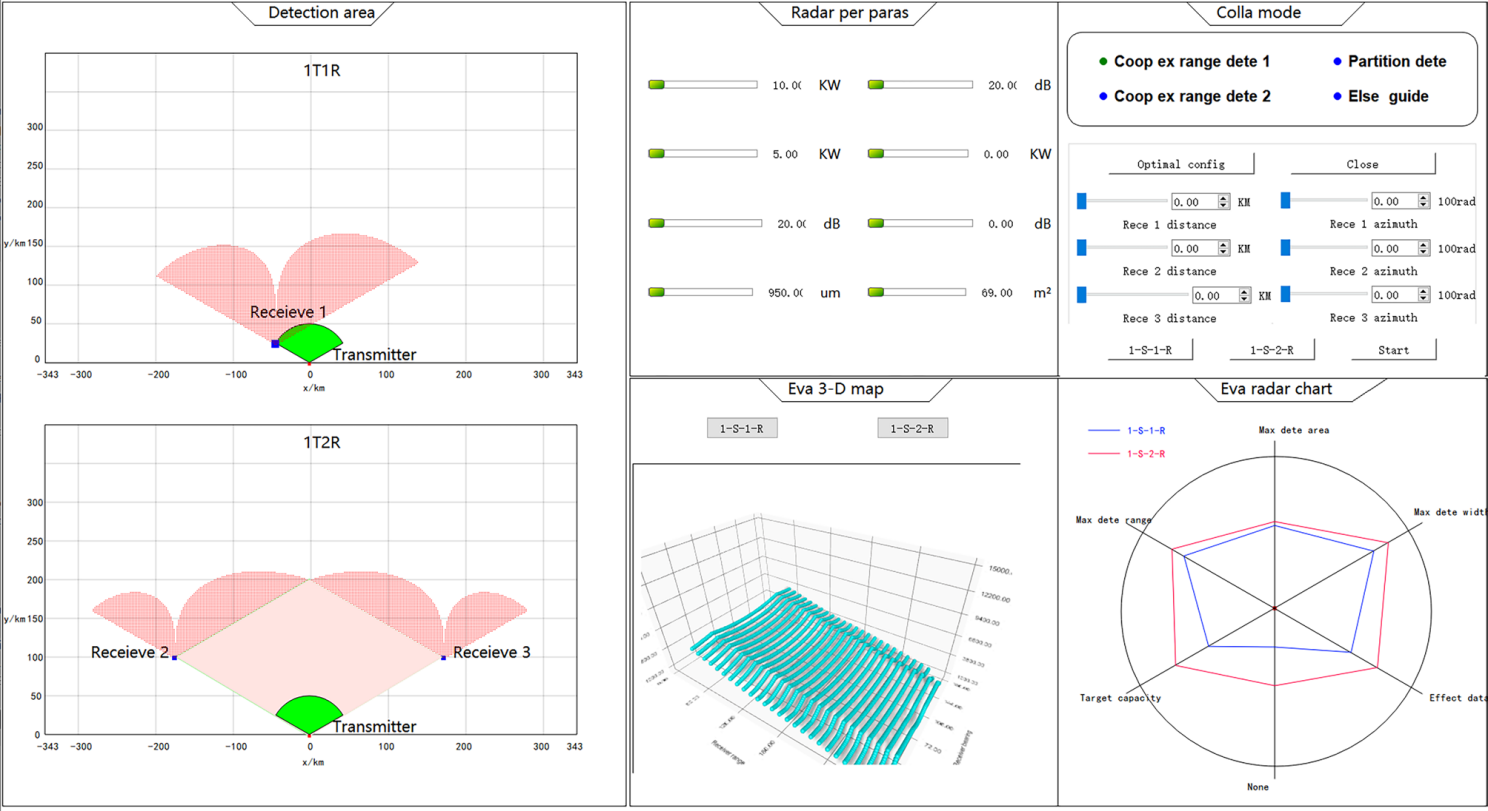
Interface for Multi-aircraft Collaborative Detection Simulation and Verification Software Developed on QT.

**Figure 12:**
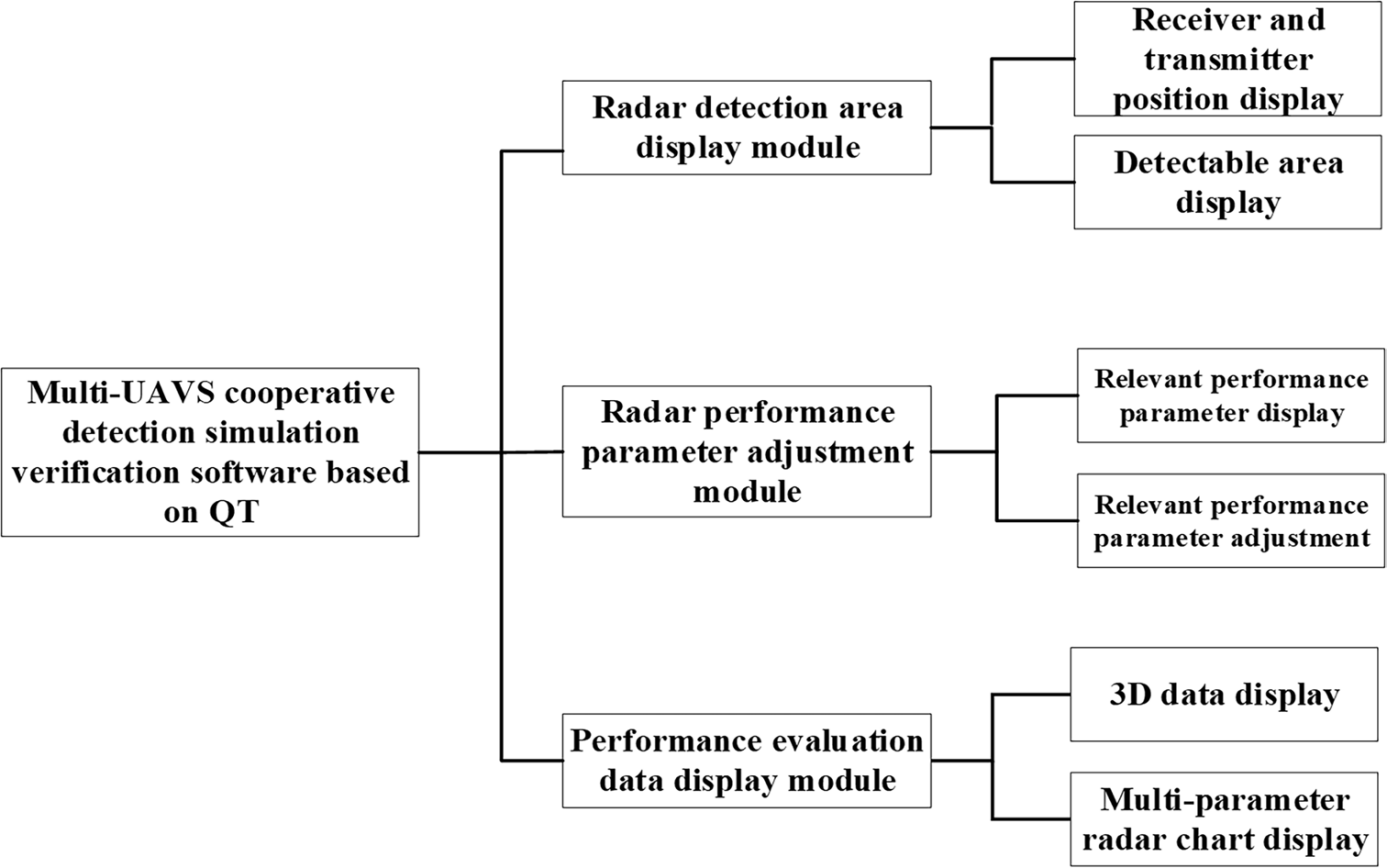
Introduce for the functional composition of a multi-machine collaborative detection simulation and verification software system developed using QT.

To validate the effectiveness of the proposed algorithm, a multi-aircraft collaborative detection semi-physical simulation verification platform was independently developed. The software component was implemented using Qt Creator 4.11.1 (Community) and OGRE engine, and the graphical user interface is illustrated in Fig. 11. The functional components of the software system are depicted in Fig. 12.

1. Radar detection zone display module.

This module provides visualization of the locations of the receivers and transmitters in a one-to-many communication scenario, along with their corresponding detection areas. By utilizing the selection buttons located on the right-hand side, the interface can display detection results for different combinations of receiver and transmitter quantities.

2. Module for Adjusting Radar Performance Parameters.

This module displays parameters related to the overall detection performance, along with corresponding sliders. By adjusting the sliders, the internal performance parameters of the program are updated accordingly, resulting in changes to the displayed detection area in the radar detection area display module.

3. Module for Performance evaluation data display.

This module presents real-time data on the size fluctuations of various evaluation metrics used to assess the overall detection efficiency.

### Validation platform validation of the effectiveness

**Figure 13:**
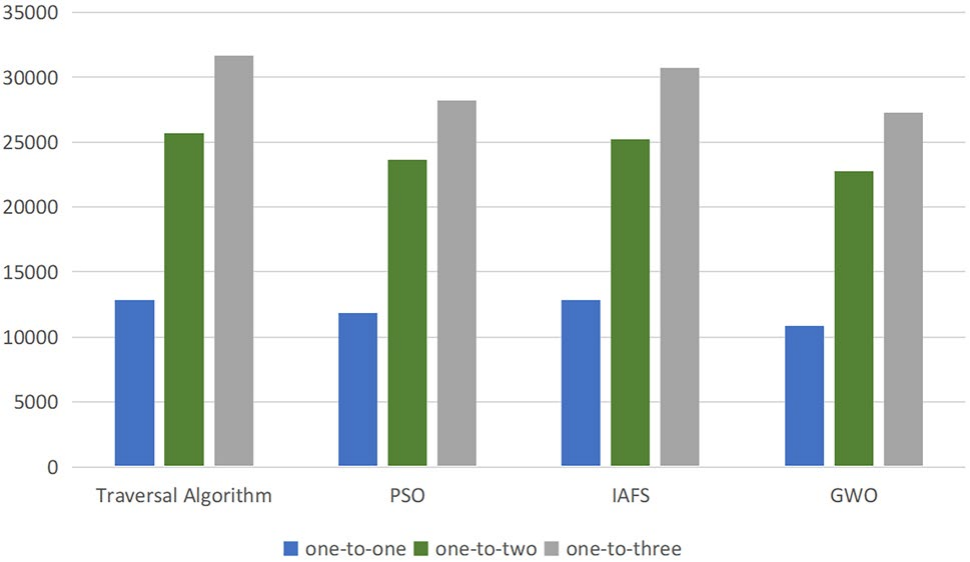
Comparison of the detection areas of the optimal configurations corresponding to the three algorithms.

Initially, to facilitate later comparisons with the optimal configurations obtained using various techniques, we randomly selected receiver positions for demonstration purposes. As depicted in Fig. 13, changes in the configuration of the separate transmitter and receiver system result in variations in the detection area. So, reasonable formation configuration settings can greatly enhance the detection efficiency.

**Figure 14:**
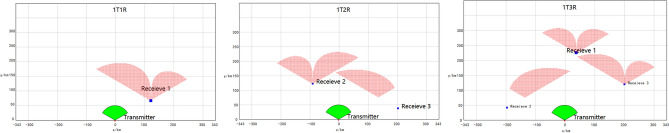
Schematic diagram of detection area under different random configurations.

**Figure 15:**
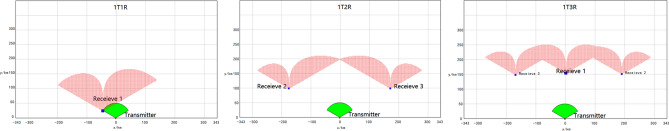
The optimal configuration diagram obtained through the application of the traversal algorithm.

**Figure 16:**
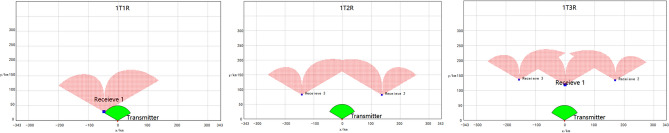
The optimal configuration diagram obtained through the application of PSO.

**Figure 17:**
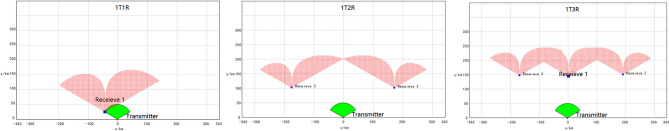
The optimal configuration diagram obtained through the application of IAFS.

**Figure 18:**
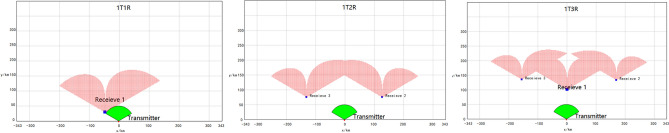
The optimal configuration diagram obtained through the application of GWO.

Next, we will apply three different algorithms: traversal algorithm, particle swarm optimization algorithm, and improved Artificial Fish Swarm Algorithm to calculate the optimal formation configuration. The results of solving one-to-one, one-to-two, and one-to-three correspond to the three methods and are shown in Figs. 14, 15, 16, 17 and 18. The y axis shows the size of the detection area.

By comparing the simulation results and the detection area comparison diagram of the optimal configurations corresponding to the three algorithms, it can be observed that there is still some deviation between the optimal formation configuration obtained by the particle swarm optimization algorithm and that obtained by the traversal algorithm. However, the optimal formation configuration obtained by the improved Artificial Fish Swarm Algorithm is almost identical to that obtained by the traversal algorithm.

Since the particle swarm optimization algorithm adopts a random search strategy and the particle search process is random without the need to adhere to rules, the obtained solutions are more random and unstable. In the testing process, there were instances where there were significant differences in the results obtained. In contrast, the improved Artificial Fish Swarm Algorithm is more accurate and stable.

In summary, the improved Artificial Fish Swarm Algorithm adopted in this study demonstrates practical usefulness due to its accuracy and stability.

**Figure 19:**
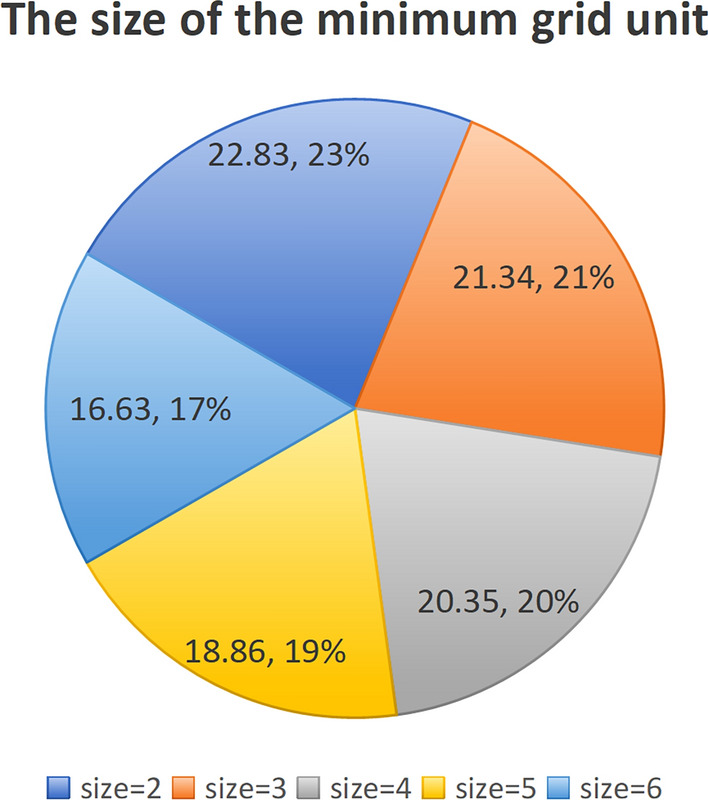
The schematic representation of the ratio between the optimal configuration corresponding to the detection area and the size of the minimum grid unit determined by the edge length of different alert zone partitions.

By observing the comparison data in Fig. 18 and the efficacy diagram from “one transmission and one reception” to “one transmission and three receptions” in Fig. 19, it can be inferred that an increase in the number of receivers results in a larger overall defense area, while keeping the transmitter constant. However, during experimentation, it was found that beyond a certain point, in this case, “one transmission and five receptions,” the increase in defense area was no longer significant. Therefore, in practical scenarios, it is necessary to choose the appropriate number of receivers to achieve ideal defense and detection effects while avoiding unnecessary resource waste.

Moreover, during the experimental process, it was discovered that the accuracy of the optimal configuration calculation was influenced by the size of the minimum grid unit used for dividing the surveillance airspace. In situations where the conditions allow, a smaller size of the grid unit results in more precise calculation outcomes. The specific comparison is shown in Fig. 19 below:

In conclusion, the validity of the proposed method has been verified through experimental analysis as presented above.

### Validation of the efficiency

**Figure 20:**
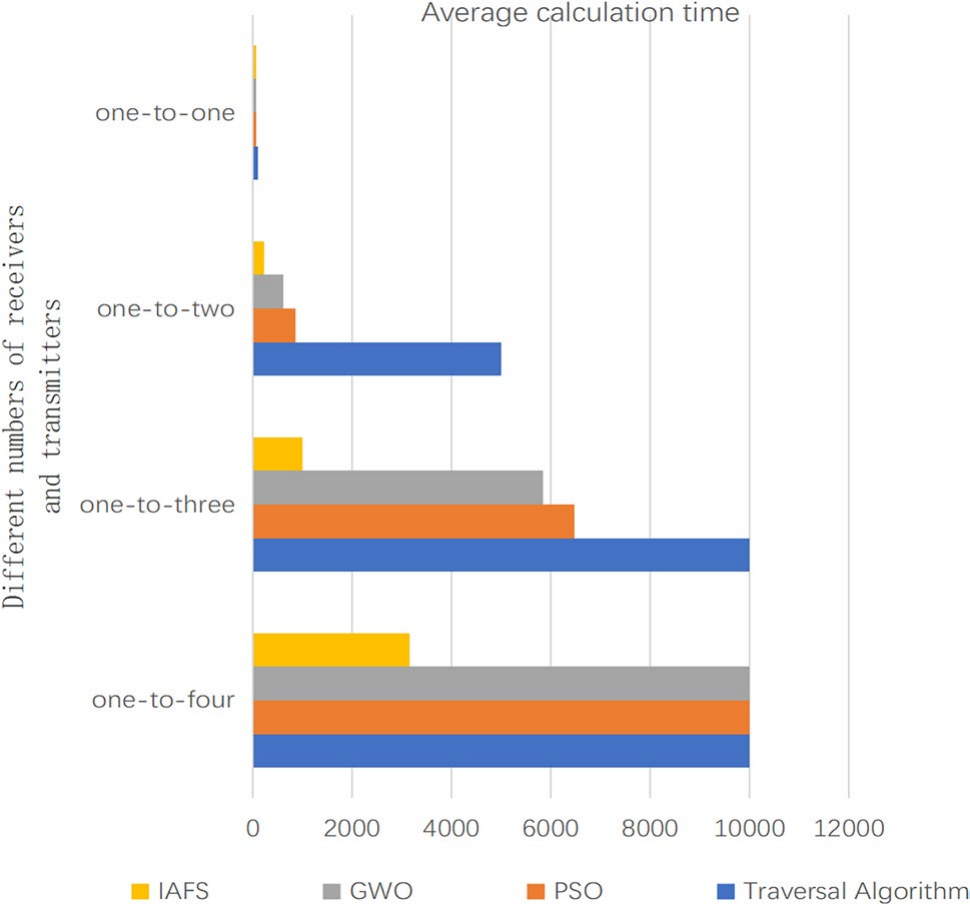
The present study provides a comparative analysis of the average computational time for optimal configurations of four algorithms, corresponding to varying quantities of transmitted and received data, as depicted through a graph.

To demonstrate the superior computational efficiency of the improved Artificial Fish Swarm Algorithm (IAFS) over Particle Swarm Optimization (PSO) in solving optimal configuration problems, we conducted the following experiments: Specifically, we employed a brute-force traversal approach, along with the IAFS ,GWO and PSO algorithms, to optimize the “one-to-one”, “one-to-two”, “one-two–three” and “one-two-four” formation configurations. Each algorithm was run 15 times, and the results were recorded. The average time cost of 15 iterations is depicted in Fig. 20.

By comparing and analyzing simulation results, we conclude that the improved Artificial Fish Swarm Algorithm can obtain the optimal solution faster. Additionally, during testing, it was observed that the improved algorithm exhibited less fluctuation in the 15 run times, indicating better stability and faster speed. In contrast, particle swarm optimization algorithm, which adopts a random search strategy, produces more random solutions and exhibits lower stability. The improved Artificial Fish Swarm Algorithm successfully avoids this phenomenon.

We conducted an analysis of the time complexity for each algorithm. The time complexity of the traversal algorithm is given by $O(W^{3} )$, where $W$ represents the number of grids that divide the entire battlefield. It is worth noting that this algorithm exhibits a significantly high computational complexity.

Furthermore, the improved Artificial Fish Swarm Algorithm exhibits a time complexity of $O\left( {\left( \frac{p}{n} \right)^{n} } \right)$, where $p$ denotes the number of individuals in the initial artificial fish population, and $n$ represents the number of receivers in the optimal configuration of the “one transmitter-many receivers” scenario to be computed.

In the case of other algorithms, such as the particle swarm optimization algorithm, the time complexity is $O(C_{p}^{n} )$. Although the differences are not prominent for small-scale scenarios, the discrepancies in algorithmic complexity become increasingly apparent as the scale of the problem grows.

A comparative illustration of the time complexities for each algorithm is depicted in Table 1.

**Table 1:** The algorithm complexity of each algorithm is compared.

Time complexity comparison			
Algorithm name	Traversal algorithm	IFSA	PSO & GWO
Time complexities	$O(W^{3} )$	$O\left( {\left( \frac{p}{n} \right)^{n} } \right)$	$O(C_{p}^{n} )$
P = 30, N = 3, W = 2000	8,000,000,000	1000	24,360
P = 30, N = 4, W = 2000	16,000,000,000,000	3164	657,720
P = 60, N = 4, W = 2000	16,000,000,000,000	50,625	11,703,240

### Validation of the practicality

To validate the effectiveness of the algorithm, a joint simulation was conducted using a self-developed data-driven 2D battlefield situation visualization based on the OGRE engine and a multi-machine collaborative detection simulation software developed using QT. The algorithm simulation test platform utilized in this study serves as both a testing platform and an implementation platform for the algorithms. These two aspects are inseparable, as there is considerable data interaction between the algorithm's specific implementation process and the testing platform. Various programming concepts, such as data structures, algorithms, and the application of multithreading, are employed during the algorithm’s implementation. Even the slightest changes in software components, such as buttons or sliders, can have an impact on certain parameters within the algorithm.

Section B of the simulation validation part in this paper provides a comprehensive overview of the overall functionality of the testing platform. The simulation platform was constructed using Qt Creator 4.11.1 (Community) software and the OGRE engine. The QT software was utilized for the overall interface design and algorithm implementation, while the two-dimensional battlefield view was incorporated using the OGRE engine. This view allows for an aerial perspective to observe the two-dimensional flight trajectories of each aircraft. Additionally, the platform includes a large-scale aircraft simulator and four small-scale aircraft simulation devices. The visual perspectives of the multiple aircraft can be synchronized. In Section E of the simulation validation part, the transmitter is operated by the large-scale aircraft simulator, while the three receivers are simulated by internally designed virtual unmanned aerial vehicles. The enemy unmanned aerial vehicles are simulated by the small-scale aircraft simulation devices. The initial information was set up for the combat scenario as per the specifications outlined in section “Background hypothesis”, as depicted in Table 2.

**Table 2:** Initialization data for mission information.

Troop name	Serial number	Longitude	Latitude	Height (/m)	Speed (m/s)	Course (/°)
Our forces	Our No. 1 plane	123.60	41.40	3000.0	500	0
	Our No. 2 plane	122.80	41.40	3000.0	500	0
Enemy force	Enemy No. 1 plane	122.90	43.427	3000.0	500	− 180
	Enemy No. 2 plane	124.00	43.3	3000.0	500	− 180

**Figure 21:**
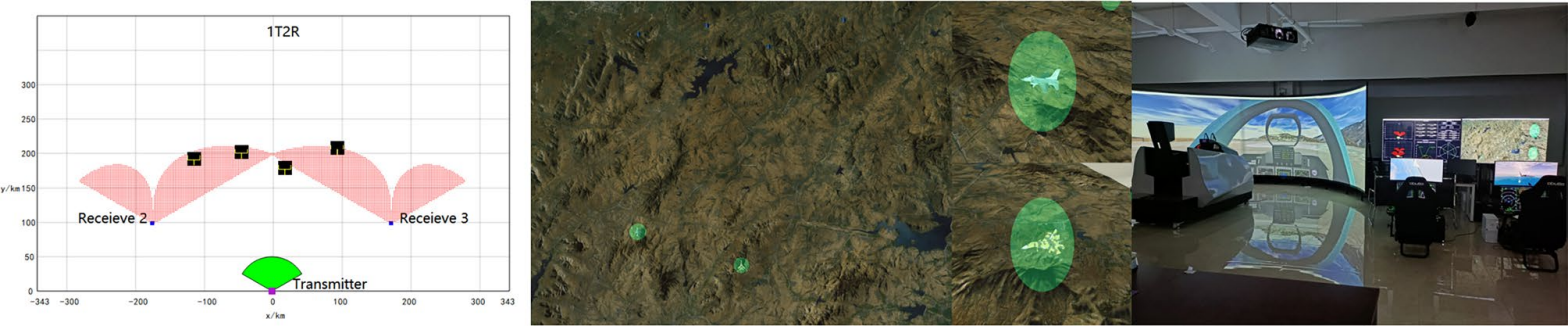
Effectiveness display of semi-physical simulation experiments.

**Figure 22:**
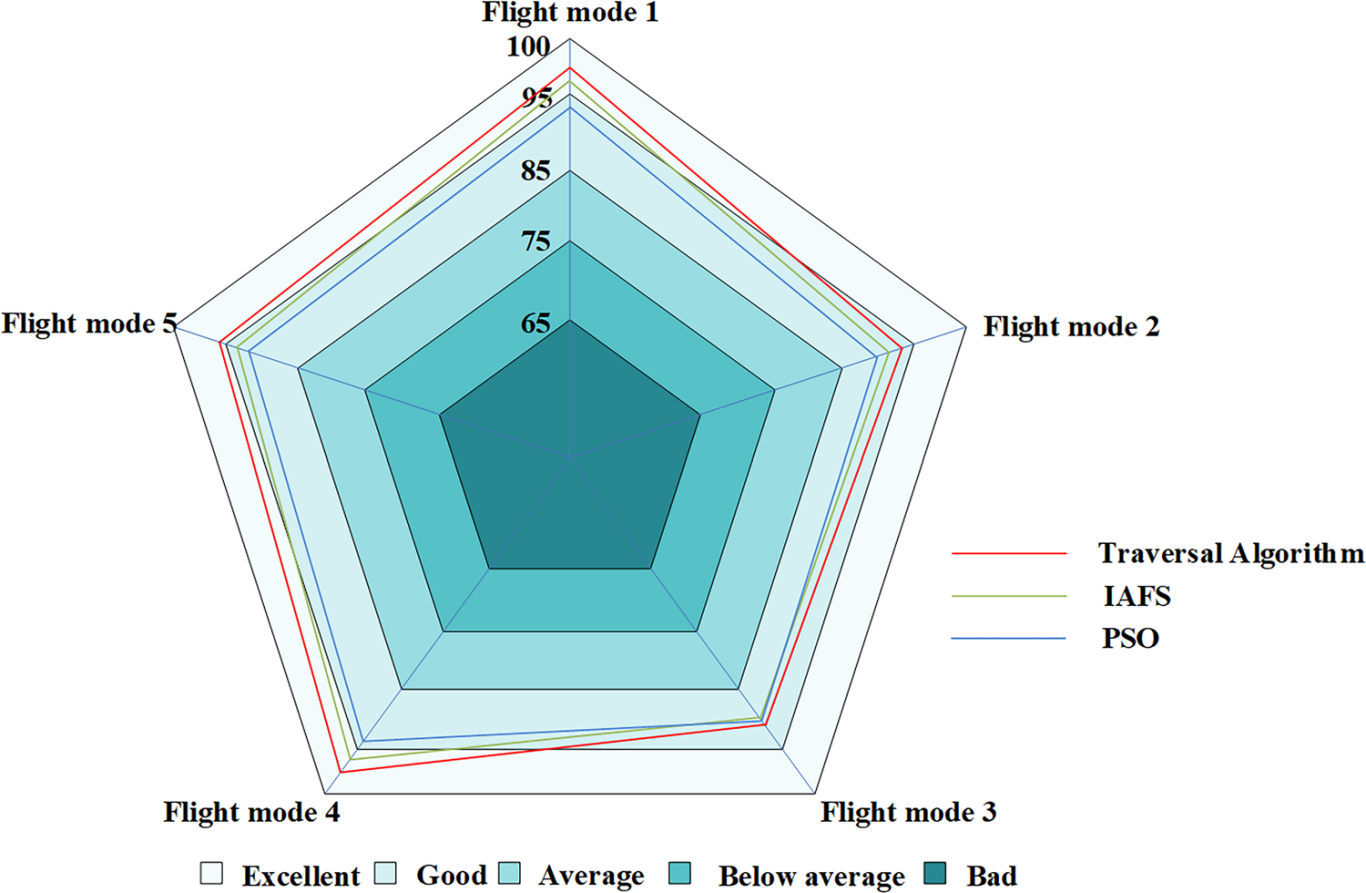
Presentation of Results from a Semi-Physical Simulation Experiment.

A multi-aircraft cooperative detection simulation software was developed using the QT framework and executed on a master computer. The red team's aircraft, which were utilized for detection purposes, followed the one-to-many transmission mode and utilized an improved Artificial Fish Swarm Algorithm for cooperative detection. The aircraft initiated their flight from the starting position and moved forward until the optimal configuration was reached, while the blue team's aircraft were controlled by a human control platform. The blue team attempted to approach the red team using different formations, but upon entering the detection area of the optimal configuration, they were all detected by the red team's aircraft. This result confirmed the practicality and effectiveness of the proposed algorithm. The experimental equipment used is shown in Fig. 21 below:

Additionally, under the scenario of one launcher and three receivers, during the process of controlling the blue aircraft towards the red side using a manned-unmanned teaming control platform, various dual-formation configurations were attempted. After multiple tests, the optimal formation configuration calculated through distinct algorithms exhibited a different detection effect on the blue aircraft, The specific comparison is shown in Fig. 22 below:

Upon analyzing the aforementioned findings, it was observed that the traversal algorithm exhibited superior detection capabilities against the opponent in comparison to the other two algorithms. Additionally, the overall performance of the improved Artificial Fish Swarm Algorithm was comparable to that of the traversal algorithm, with the former demonstrating better efficacy in the majority of cases when compared to the particle swarm algorithm. Notably, the particle swarm algorithm displayed a high degree of randomness, resulting in unstable configurations. Furthermore, it was observed that the majority of outcomes were inferior to those produced by the improved Artificial Fish Swarm Algorithm.

During the process of collaborative detection in an aircraft cluster, the number and types of participating aircraft may vary dynamically. When the number of aircraft changes while the aircraft types remain the same, it is only necessary to recalculate the optimal formation configuration for different quantities. In the event of changes in aircraft types, modifying the radar parameters in the calculation process and subsequently considering the issue of the number of aircraft within the formation is sufficient. To reduce the computational burden of determining the optimal formation configuration during aircraft movement, it is possible to pre-establish a library of optimal formation configurations by calculating the configurations for different aircraft types and quantities in advance. By utilizing the algorithm proposed in this paper, both real-time calculation of optimal formation configurations and the pre-design of a library demonstrate practical value while maintaining the accuracy and stability of the algorithm. Further practical testing with actual aircraft is required to validate these concepts and provide valuable insights for future technological advancements.

In conclusion, the proposed improved Artificial Fish Swarm Algorithm was employed to optimize the configuration of the "one-to-many" airborne radar network with separated transmitting and receiving functions for collaborative target detection. The results demonstrated that the algorithm achieved superior solutions with shorter computation times compared to other methods. Moreover, the algorithm partially overcame the limitation of unstable performance that is often observed in intelligent algorithms. These findings further validate the potential of the separated transmitting and receiving mode in airborne radar networks for target detection and provide a foundation for future research on clustered collaborative detection configurations.

## Conclusion

Considering the spatial variations of aircraft scatter radar waves, this study investigates an optimization approach for determining the optimal configuration of a space-based radar cooperative detection formation using an improved Artificial Fish Swarm Algorithm.

The main conclusions regarding the algorithm improvements are as follows:

In our study, we have identified several key differentiators and improvements in our proposed optimization method compared to the existing improved AFSA. These identifications are as follows:

1. We have identified a more efficient update mechanism for the solution search process in our method. By incorporating adaptive adjustment strategies, our algorithm achieves faster convergence while maintaining a balance between exploration and exploitation.
2. By combining the expert knowledge in this field, the optimization method proposed by us modifies the rule-based search strategy for the improved AFSA, and the three typical behaviors within it are also customized and optimized for the content of this study. These strategies enhance the exploration and development ability of algorithms, thus improving the quality of convergence and resolution.
3. Our proposed method addresses a specific limitation in the existing improved AFSA related to the handling of dynamic optimization problems. We have identified and implemented a mechanism that enables our algorithm to dynamically adapt its search behavior based on the changing number of receivers, resulting in improved performance in dynamic environments.

These identifications highlight the unique contributions and advancements of our proposed optimization method compared to the existing improved AFSA. We believe that these differentiators enhance the algorithm's capabilities, leading to improved convergence speed, solution quality, and adaptability in various problem scenarios.

Regarding the data comparison with other algorithms:

1. In terms of algorithm efficiency, taking the “one transmission and three receptions” scenario as an example, the proposed IFSA algorithm exhibits improvement percentages of approximately 90.18%, 82.88%, and 84.56% compared to the exhaustive search, GWO, and PSO algorithms, respectively.
2. In terms of the effectiveness of the computational results, using the “one transmission and three receptions” scenario as an example, the proposed IFSA algorithm demonstrates improvement percentages of approximately 12.68% and 9.12% compared to the GWO and PSO algorithms, respectively.

Please note that the percentages mentioned above are approximate values.

The primary conclusions regarding the overall study are as follows:

1. An optimization mathematical model was developed for the configuration of space-based radar networks, which aimed to optimize defense coverage area, transmitter and receiver safety, and detection efficiency. To achieve this, optimization evaluation indicators were selected and used to establish a collaborative detection configuration for the receiving and transmitting components of the radar network. The model was designed to optimize the spatial formation of the network and promote the effective coordination of its components.
2. We conducted a simulation analysis of the “One transmitter, multiple receivers” random formation configuration and the optimal formation configuration based on an improved Artificial Fish Swarm Algorithm for cooperative detection of air-based radar network with separated transmitting and receiving systems. The simulation results demonstrate that optimizing the spatial configuration of the transmitter and receiver can effectively leverage the advantages of cooperative detection in air-based radar network with separated transmitting and receiving systems, thereby improving the detection performance of the target and enhancing the survivability of the host.

The presented research methodology in this study provides a valuable reference for various practical applications in the context of aircraft swarm operations, such as task planning, tactical deployment, and collaborative strike. Furthermore, by incorporating situational analysis in specific battlefield scenarios, we can develop a strategic decision-making framework for collaborative detection and engagement, which can effectively address corresponding situations in aerial warfare and provide robust technical support for the advancement of multi-aircraft collaborative combat technology.

## Data Availability

Te datasets generated during and/or analysed during the current study are available from the corresponding author on reasonable request.
